# Observational and Genetic Association of Myelodysplastic Syndromes (MDS) and Autoimmune Diseases in Adults

**DOI:** 10.1155/mi/3088404

**Published:** 2026-02-17

**Authors:** Qizhao Li, Yujin Guo, Gao Xiao, Xuejing Song, Yuying Wei, Wenjuan Gao, Gege Feng, Xinyi Zuo, Xue Shi, Hongyu Zhao, Yuefen Hu, Johan Rebetz, Elisabeth Semple, Li Guo, John W. Semple, Jun Peng, Shuqian Xu

**Affiliations:** ^1^ Department of Hematology, Qilu Hospital of Shandong University, Jinan, Shandong, China, qiluhospital.com; ^2^ Department of Ophthalmology, The Second Hospital of Jilin University, Changchun, China, jlu.edu.cn; ^3^ Department of Vascular Surgery, Shandong Provincial Hospital Affiliated to Shandong First Medical University, Jinan, Shandong, China, sph.com.cn; ^4^ Jinan Vocational College of Nursing, Jinan, Shandong, China; ^5^ Department of Hematology, The Affiliated Hospital of Qingdao University, Qingdao, China, qdu.edu.cn; ^6^ Department of Hematology, Jinan Central Hospital Affiliated to Shandong First Medical University, Jinan, Shandong, China; ^7^ Department of Hematology, Shandong Provincial Hospital Affiliated to Shandong First Medical University, Jinan, Shandong, China, sph.com.cn; ^8^ Division of Hematology and Transfusion Medicine, Lund University, Lund, Sweden, lu.se; ^9^ Bloodworks Northwest Research Institute, Seattle, Washington State, USA; ^10^ Division of Hematology and Oncology, University of Washington, Seattle, Washington State, USA, washington.edu; ^11^ Institute for Stem Cell and Regenerative Medicine, University of Washington, Seattle, Washington, USA, washington.edu; ^12^ Clinical Immunology and Transfusion Medicine, Office of Medical Services, Region Skåne, Lund, Sweden, skane.se; ^13^ Departments of Pharmacology, Medicine and Laboratory Medicine and Pathobiology, University of Toronto, Toronto, Canada, utoronto.ca; ^14^ Shandong Provincial Clinical Research Center for Hematological Diseases, Jinan, Shandong, China; ^15^ Shandong Key Laboratory of Hematological Diseases and Immune Microenvironment, Jinan, Shandong, China

**Keywords:** autoimmune diseases, bioinformatic analysis, immune cells, Mendelian randomization, multicenter retrospective study, myelodysplastic syndrome

## Abstract

About 25% of patients with myelodysplastic syndromes (MDS) have combined autoimmune diseases (AIDs). However, the relationships between MDS and AIDs, especially a causal relationship and the underlying shared pathophysiological mechanisms, remain largely unknown. We aimed to evaluate the association between MDS and AIDs using a multicenter retrospective study, Mendelian randomization (MR), and bioinformatics analysis. About 26.6% of patients with MDS from all centers presented with AIDs. Compared to MDS patients without AIDs, MDS with AIDs was less likely to progress to acute myeloid leukemia (AML) (6.6% vs. 15.1%, *p* = 0.037), and the pre‐existing AIDs could be used as an independent protective factor of survival (HR: 0.504, *p* = 0.048). Bidirectional MR results showed that MDS could cause the risk of systemic lupus erythematosus (SLE, OR: 1.09, *p* = 0.015), although with no significant causal relationship in other AIDs. The effect of MDS on SLE may be partially mediated by naïve CD4+ T‐cells (median proportion 6.9%) and CD45RA‐CD4+ T memory cells (median proportion 9.0%). Furthermore, two hub genes (IFI27 and VSIG4) were identified by machine learning and curve analysis as potential diagnostic markers for MDS with SLE. Our study suggested that the impact and mechanisms of AIDs in MDS need to be taken seriously, which could provide more accurate treatment guidance.

**Summary**

• Integrating data from four Chinese centers, a comprehensive view of the association between MDS and autoimmune diseases (AIDs) was provided. AIDs lower the incidence of leukemia in MDS patients, and especially, the pre‐existing AIDs could be used as an independent protective factor for MDS patients.
• MR analysis provided the first insight into a direct causal relationship between MDS and AIDs. The role of specific immune cell subsets (naïve CD4+ T and CD45RA‐CD4+ T memory cells) in MDS‐induced SLE was revealed for the first time, which provides a new mechanism for understanding the interaction between MDS and AIDs.
• Univariate COX regression analysis suggested that following AIDs (>1 year after MDS) was a risk of survival; thus, we identified common diagnostic markers (IFI27 and VSIG4) and shared pathological processes between MDS and SLE for the first time, which provides insights for preventing or monitoring the occurrence of AIDs in MDS patients.

## 1. Introduction

Myelodysplastic syndromes (MDS) are a group of malignant clonal disorders affecting bone marrow hematopoietic stem and progenitor cells, which are characterized by ineffective and pathological hematopoiesis and high risk of transformation to acute myeloid leukemia (AML) [1]. In recent years, a growing number of observational studies reported that 10%–30% of MDS patients have combined autoimmune diseases (AIDs), especially rheumatoid arthritis (RA), hypothyroidism, systemic lupus erythematosus, and psoriasis [2–4]. Anderson et al. [5] and Kristinsson et al. [6] noted that pre‐existing AIDs were associated with an elevated risk of MDS (OR: 1.5) based on the SEER database. However, Al Ustwani et al. [7] and Kim et al. [4] reported that MDS has a higher risk for AIDs. It is suspected that the heterogeneity of MDS pathogenesis or treatment may contribute to the development of AIDs [8, 9]. Indeed, persistent immune imbalances (such as TGF‐β pathway [10, 11] and T‐cell homeostasis [12]) have been reported in early‐stage or low‐ to intermediate‐risk MDS patients, and these long‐term abnormal immune or inflammatory responses can contribute to the development of AIDs. On the other hand, several studies suggested that MDS patients with AIDs may have a lower rate of progression to AML and better survival [8, 13]. Conversely, Adrianzen‐Herrera et al. [2] found that AIDs in low‐risk MDS is instead a risk factor for the progression to AML. The study [7] also suggested that MDS patients with AIDs have shorter survival. It appears that the prognostic impact of AIDs on MDS is controversial, and it is still unclear whether there are direct associations (especially causality) and underlying mechanisms between AIDs and MDS [2, 3, 13]. Thus, it is essential to explore the evidence and significance of these associations based on clinical or basic mechanistic research.

Mendelian randomization (MR) [14] is based on genetic variation as instrumental variables (IVs) to infer causality between exposure and outcome. It is an effective tool to overcome reverse causation, confounding, and bias in observational studies. Furthermore, bioinformatics technology can provide a comprehensive analysis of the potential interrelationships in diseases.

For these reasons, we utilized a multicenter retrospective study, mediation MR, and bioinformatics methodology for exploring the relationship and shared pathological biomarkers between MDS and AIDs.

## 2. Materials and Methods

### 2.1. Study Design

**Figure 1:**
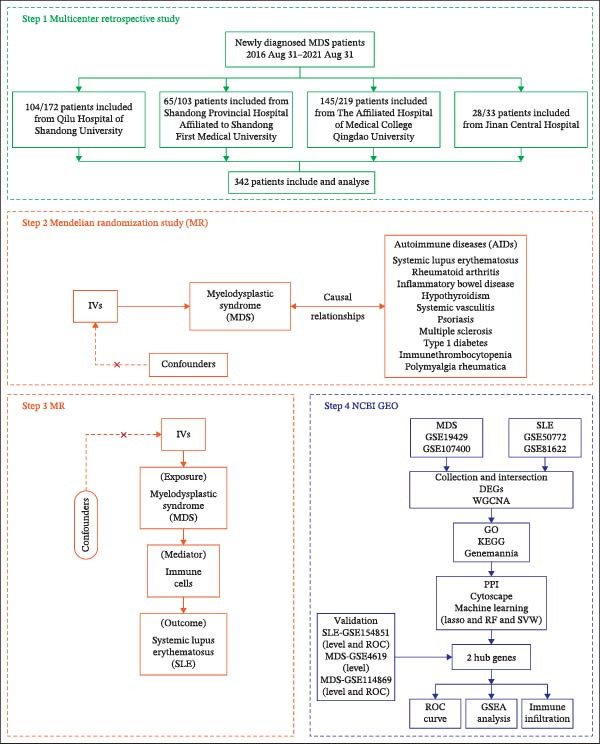
Study flow. Based on the multicenter retrospective study, the patient clinical baseline between MDS and AIDs or SI# was described, as well as the analyzed impact of AIDs or SI# on the progression to AML and survival of MDS patients (Step 1). Two‐sample MR analysis (TSMR) of the causality and direction of association between MDS and 10 types of AIDs (Step 2). Two‐step MR analysis of the mediating role of dysregulated immune cells between MDS and SLE (Step 3). Bioinformatics techniques were used to explore the underlying pathophysiological mechanisms of MDS and SLE (Step 4).

First, based on a multicenter retrospective observational study, we analyzed the correlation between MDS and AIDs, as well as their impact on the progression to AML and survival of MDS patients. A two‐sample MR analysis (TSMR) was then used to determine the causality and direction of association between MDS and 10 types of AIDs. Further, a two‐step MR analysis was utilized to reveal the mediating role of dysregulated immune cells between MDS and SLE. The MR research adhered to the Strengthening the Reporting of Observational Studies in Epidemiology using Mendelian Randomization (STROBE‐MR) guidelines (Supporting Information 1). Finally, the underlying pathophysiological mechanisms of MDS and SLE were explored based on bioinformatics techniques. Figure 1 shows the flowchart of the study design and analysis process.

### 2.2. Patients and Data Source

We retrospectively collected clinical data of patients with the initial diagnosis of MDS between August 31, 2016, and August 31, 2021, and followed up until December 31, 2023, at four centers in China. Patients were classified according to the WHO 2016 criteria for MDS [15] and AIDs usual international classification criteria [16–19]. MDS patients were excluded if they were younger than 18 years of age; had other hematologic neoplastic diseases; had experienced chemotherapy before diagnosis; had only positive serology; or had incomplete medical records. Furthermore, we divided AIDs into three groups according to the AIDs diagnosis time: the pre‐existing AIDs (>1 year before MDS); the timely associated (within 1 year before or after MDS diagnosis); and the following AIDs (>1 year after MDS). The study was approved by the Research Ethics Committee of Qilu Hospital of Shandong University (Approval No.: KYLL‐202405‐053).

Genome‐wide association studies (GWAS) summary dates for 10 types of AIDs. MDS and polymyalgia rheumatica’s dates from FinnGen (https://www.finngen.fi/en). These databases are based on European populations and from independent cohorts. The Gene Expression Omnibus (GEO database, https://www.ncbi.nlm.nih.gov/geo/) [20] was used for bioinformatic analysis. Details are shown in Supporting Information 2. Since these databases are based on publicly available summary‐level data, they do not require ethical approval.

### 2.3. MR Analysis

In this study, we initially adopted the conventional genome‐wide significance threshold of *p* < 5 × 10^−8^ for the selection of IVs, a standard widely applied in MR research. However, for MDS and certain AIDs, the number of single nucleotide polymorphisms (SNPs) meeting this stringent threshold was limited, making it difficult to ensure sufficient statistical power and reliable MR inference. To address this issue, we referred to previous MR studies on MDS and related diseases and relaxed the threshold to *p* < 5 × 10^−6^ (Supporting Information 2) [21, 22]. This approach has been employed in multiple studies as a common strategy when the number of available IVs is insufficient, and its validity has been supported by empirical evidence [23, 24].

For mediation analysis, we adjusted the significance level based on the counts of selected SNPs exceeding 20. Subsequently, we used a clustering strategy with a threshold of *r* ^2^ <0.001 and kb = 10,000 to reduce linkage disequilibrium (LD). Palindromic SNPs as well as SNPs strongly associated with the outcome were excluded. Finally, to assess the strength of IVs, we calculated the *F*‐statistic, and only SNPs with *F*‐statistics >10 were retained for subsequent MR analysis to avoid bias from weak IVs.

Inverse‐variance weighted (IVW) was applied as the primary MR analysis method. To improve the accuracy of the results, we also used MR‐Egger, weighted median, simple mode, and weighted mode for additional analysis. We performed sensitivity analysis to assess potential heterogeneity and horizontal pleiotropy. Cochran’s *Q* test was used to detect heterogeneity between estimates of SNPs. When heterogeneity existed, a random effects IVW model was selected; otherwise, a fixed effects IVW model was used. The MR‐Egger intercept test, the MR‐PRESSO (MR‐Pleiotropy ReSidual Sum and Outlier), and the global test were used to test for horizontal pleiotropy. In addition, leave‐one‐out analysis was performed, and scatterplots and funnel plots were drawn to visualize the results.

To investigate potential immune‐mediated pathways between MDS and SLE, we performed a two‐step MR analysis to explore the mediating role of 731 immune cell phenotypes. First, we identified immune cell phenotypes with a significant causal effect (*p* FDR < 0.05) on SLE by TSMR. Subsequently, we assessed the effect of MDS on immune cells statistically significantly associated with SLE and also by TSMR. The overall effect of MDS on SLE can be categorized into direct and indirect effects. Indirect effects were quantified using the “product of coefficients” method. The proportion of the total effect mediated by immune cells is determined by dividing the indirect effect (β1×β2) by the total effect (β3). Here, β1, β2, and β3 represent the effect of MDS on immune cells, the effect of immune cells on SLE, and the effect of MDS on immune cells, respectively. In addition, standard errors (SEs) and confidence intervals were calculated using the “delta method.” The above analyses were performed using the “TwoSampleMR” (version 0.5.8), “MendelianRandomization” (version 0.9.0), and “MR‐PRESSO” (version 1.0) packages.

### 2.4. Bioinformatics Analysis

Differentially expressed genes (DEGs) and disease‐associated genes were identified by the R package “limma” and “Weighted gene co‐expression network analysis (WGCNA).” Venn diagrams were used to screen genes (CGs) in the MDS and SLE. Gene enrichment analyses (GO/KEGG) were further performed based on “clusterProfiler,” as well as gene interaction and potential function analysis relying on the GeneMANIA database (http://genemania.org/). After being screened by the String database (https://string-db.org/), Cytoscape, cytoHubb, and machine learning methods, the potential diagnostic biomarkers were identified, and their diagnostic efficiency was assessed using receiver operating characteristic (ROC) curves and the area under the ROC curve (AUC) values. The predictive diagnostic efficiency and consistency of the biomarkers were analyzed by internal and external validation, and gene set enrichment analysis (GSEA) was used to view the degree of biomarker‐associated gene set enrichment. Finally, the distribution of immune cells between disease and normal groups was explored using the cell‐type identification by estimating relative subsets of RNA transcripts (CIBERSORT) algorithm.

### 2.5. Statistical Analysis

The retrospective study was analyzed by SPSS (version 27). Differences between the groups were assessed by Pearson’s chi‐squared test or Fisher–Freeman–Halton exact test for categorical variables and the Student’s *t*‐test for continuous variables. The survival of MDS patients was tested by the COX regression model, the Kaplan–Meier method, and the log‐rank test. MR and bioinformatics correlation analyses were statistically analyzed and visualized using R (version 4.2.3). *p*‐Values were adjusted by FDR adjustment in MR for multiple hypotheses, and when *p*  < 0.05 indicated a significant difference in results.

## 3. Results

### 3.1. The Relationships and the Impact of AIDs on MDS Based on Retrospective Study

#### 3.1.1. Clinical Baseline of MDS Patients

A total of 342 MDS patients (136 females and 206 males) in four centers were included, and 91 MDS patients (26.6%) presented with AIDs. The characteristics of MDS for categorization, karyotype abnormalities, IPSS‐R, and IPSS were variable in individuals with AIDs (Table 1 and Supporting Information 3A). The most common subtype of MDS patients with AIDs was MDS with multilineage dysplasia (MDS‐MLD, 37.4%). Patients with AIDs had a higher rate of karyotypic normalization compared to those without AIDs (70.3% vs. 59.8%, *p* = 0.074), and IPSS scores were more likely to be distributed in the low‐ and intermediate‐1 risk groups [low + intermediate‐1 risk (with AIDs vs. without AIDs): 70.3% vs. 57.8%, *p* = 0.105].

**Table 1:** Baseline characteristics of patients without AIDs and patients with AIDs.

Characteristic		MDS without AIDs (*n* = 251)	MDS with AIDs (*n* = 91)	*p* value
Age, mean (S.D.), years	—	58 (14)	61 (14)	0.191^a^

Female/male, *n* (%)	—	94/157 (59.9)	42/49 (46.2)	0.146^b^

WHO 2016, *n* (%)	MDS‐SLD	4 (1.6)	2 (2.2)	0.126^c^
	MDS‐MLD	74 (29.5)	34 (37.4)	—
	MDS‐EB1	60 (23.9)	14 (15.4)	—
	MDS‐EB2	75 (29.9)	21 (23.1)	—
	MDS‐RS‐SLD	8 (3.2)	4 (4.4)	—
	MDS‐RS‐MLD	16 (6.4)	5 (5.5)	—
	MDS 5q syndrome	2 (0.8)	0 (0)	—
	MDS‐U	12 (4.8)	11 (12.1)	—

Karyotype	Normal, *n* (%)	150 (59.8)	64 (70.3)	0.074^b^

Karyotype IPSS‐R	Very favorable	3 (1.2)	1 (1.1)	0.217^c^
	Favorable	163 (64.9)	71 (78.0)	—
	Intermediate	38 (15.1)	10 (11.0)	—
	Poor	19 (7.6)	3 (3.3)	—
	Very poor	28 (11.2)	6 (6.6)	—

IPSS	Low	17 (6.8)	9 (9.9)	0.105^b^
	Intermediate‐1	128 (51.0)	55 (60.4)	—
	Intermediate‐2	82 (32.7)	24 (26.4)	—
	High	24 (9.6)	3 (3.3)	—

IPSS‐R	Very low	2 (0.8)	2 (2.2)	0.145^c^
	Low	32 (12.7)	17 (18.7)	—
	Intermediate	62 (24.7)	28 (30.8)	—
	High	84 (33.5)	21 (23.1)	—
	Very high	71 (28.3)	23 (25.3)	—

AML, *n* (%)	—	38 (15.2)	6 (6.6)	0.037 ^∗^^b^

Bone marrow blasts, mean (S.D.), *n* (%)	—	6.9 (5.2)	5.7 (5.3)	0.065^a^

Death, *n* (%)	—	173 (68.8)	64 (70.3)	0.803^b^

PFS, median (IQR), months	—	24.1 (7.5,42.9)	28.8 (13.2,52.0)	0.450^e^
Follow‐up, median (IQR), months	—	26.6 (10.4,43.8)	29.5 (13.2,52.0)	0.647^e^

*Note:* *p*  < 0.05 ^∗^ means significance.

Abbreviations: AIDs, autoimmune diseases; AML, acute myeloid leukemia; IQR, interquartile range; PFS, progression‐free survival; S.D., standard derivation.

^a^Student’s *t*‐test.

^b^Pearson test.

^c^Fisher–Freeman–Halton exact test.

^e^log‐rank test.

#### 3.1.2. Characteristics of MDS Patients With Different Types of AIDs

Among the 91 MDS patients with AIDs, the top four most common diseases were hypothyroidism (15.4%, *N* = 14), psoriasis (12.1%, *N* = 11), unclassified connective tissue diseases (CTDs) (11.0%, *N* = 10), and RA (9.9%, *N* = 9) (Table 2). Among those different types of AIDs, MDS‐MLD and MDS with excess blasts‐2 (MDS‐EB2) were more prevalent, and about 2/3 of AIDs were found within 1 year before or after MDS diagnosis (the timely association).

**Table 2:** Types of AIDs in MDS subtypes.

Disease type			MDS subtype							Diagnosis AIDs		
AIDs (*n* = 91)		MDS‐SLD, *N* (%)	MDS‐MLD, *N* (%)	MDS‐EB1, *N* (%)	MDS‐EB2, *N* (%)	MDS‐RS‐SLD, *N* (%)	MDS‐RS‐MLD, *N* (%)	MDS 5q syndrome, *N* (%)	MDS‐U, *N* (%)	Pre‐existing, *N*	Timely, N	Following, N
Skin	Vitiligo (*N* = 4)	0	1 (25.0)	1 (25.0)	0	0	2 (50.0)	0	0	2	2	0
	Psoriasis (*N* = 11)	0	6 (54.5)	2 (18.2)	2 (18.2)	0	0	0	1 (9.1)	3	6	2
	SS1 (*N* = 2)	0	0	0	0	0	0	0	2	0	1	1

Thyroid	Hypothyroidism (*N* = 14)	0	7 (50.0)	2 (14.3)	4 (28.6)	0	0	0	1 (7.1)	2	10	2
	Hyperthyroidism (*N* = 3)	0	2 (66.7)	0	1 (33.3)	0	0	0	0	1	0	2
	HT (*N* = 6)	0	4 (66.7)	0	1 (16.7)	0	1 (16.7)	0	0	0	6	0

CTDs	Unclassified (*N* = 10)	0	1 (10.0)	2 (20.0)	3 (30.0)	0	2 (20.0)	0	2 (20.0)	0	8	2
	SLE (*N* = 3)	0	1 (33.3)	1 (33.3)	1 (33.3)	0	0	0	0	1	2	0
	SS2 (*N* = 5)	0	1 (20.0)	1 (20.0)	2 (40.0)	0	0	0	1 (20.0)	1	4	0
	IgG4‐RD (*N* = 1)	0	1	0	0	0	0	0	0	0	1	0

Inflammation arthritis	Unclassified (*N* = 2)	0	0	1 (50.0)	0	0	1 (50.0)	0	0	2	0	0
	AS (*N* = 4)	0	1 (25.0)	1 (25.0)	1 (25.0)	0	0	0	1 (25.0)	1	3	0
	RA (*N* = 9)	1 (11.1)	3 (33.3)	0	2 (22.2)	2 (22.2)	0	0	1 (11.1)	1	6	2
	GA (*N* = 7)	0	1 (14.3)	2 (28.6)	3 (42.9)	1 (14.3)	0	0	0	2	4	1

Systemic vasculitis	BS (*N* = 6)	1 (16.7)	3 (50.0)	0	0	0	0	0	2 (33.3)	1	5	0
	Unclassified (*N* = 2)	0	1 (50.0)	0	1 (50.0)	0	0	0	0	1	1	0
	PMR (*N* = 1)	0	0	0	1	0	0	0	0	0	1	0

Liver	ALD (*N* = 4)	0	3 (75.0)	0	1 (25.0)	0	0	0	0	1	3	0

Gastrointestinal system	CD (*N* = 2)	0	1 (50.0)	0	1 (50.0)	0	0	0	0	1	1	0
	UC (*N* = 1)	0	1	0	0	0	0	0	0	0	1	0

Blood	ITP (*N* = 1)	0	1	0	0	0	0	0	0	1	0	0
	APS (*N* = 1)	0	0	0	0	0	1	0	0	0	1	0

Sumtotal		2	39	13	24	3	7	0	9	21	66	12

*Note:* Pre‐existing MDS (>1 year before MDS), timely associated (1 year before or after MDS diagnosis); following MDS (>1 year after MDS);

Abbreviations: AIDs, autoimmune diseases; ALD, autoimmune liver disease; APS, anti‐phospholipid syndrome; AS, ankylosing spondylitis; BS, Behçet′s syndrome; CD, Crohn’s disease; CTDs, connective tissue diseases; GA, gouty arthritis; HT, hashimoto thyroiditis; IgG4‐RD, IgG4‐related diseases; ITP, immune thrombocytopenia; PMR, polymyalgia rheumatica; RA, rheumatoid arthritis; SLE, systemic lupus erythematosus; SS1, sweet syndrome; SS2, Sjogren’s syndrome; UC, ulcerative colitis.

#### 3.1.3. Prognostic and Survival Analysis

MDS patients with AIDs had a significantly lower risk of progressing to AML when compared to the MDS patients without AIDs group (6.6% vs. 15.1%, *p* = 0.037) (Table 1). Even after excluding the patient group that did not receive therapeutic intervention to eliminate its potential interference, the analysis results still remained statistically significant (Supporting Information 3B,C). Overall 70.3% of MDS patients with AIDs died during the follow‐up period, which was not significantly different from the MDS patients without AIDs group (68.8%, *p* = 0.803). The progression‐free survival (28.8 vs. 24.1, *p* = 0.450) and follow‐up (29.5 vs. 26.6, *p* = 0.647) were also similar in both groups (Table 1 and Figure 2A,B), but among the MDS patients that received therapeutic intervention, the progression‐free time of MDS patients with AIDs was significantly longer than that of MDS patients without AIDs (34.43 vs. 28.15, *p* = 0.048) (Supporting Information 3B,C). When subdividing AIDs diagnosis time, it was found that MDS patients with the pre‐existing AIDs (>1 year before MDS) had a significantly longer survival time compared to those without AIDs (52.6 vs. 26.6, *p* = 0.017). Conversely, MDS patients diagnosed with the following AIDs (>1 year after MDS) had a shorter survival time (22.4 vs. 26.6, *p* = 0.052) (Supporting Information 3 and Figure 2C,D); after excluding non‐therapeutic intervention factors that accelerate disease progression, the results remained statistically significant (Supporting Information 3B,C).

Figure 2Kaplan–Meier survival curves in MDS patients with or without AIDs. The progression‐free survival (A) and overall survival (B) of MDS patients with or without ADSI#. The progression‐free survival (C) and overall survival (D) of MDS patients without or with AIDs, and the time of diagnosis of AIDs were subdivided into subgroups. ADSI#, autoimmune diseases and systemic inflammation (except isolated ANA 1:100) and AIDs, autoimmune diseases.

**Figure:**
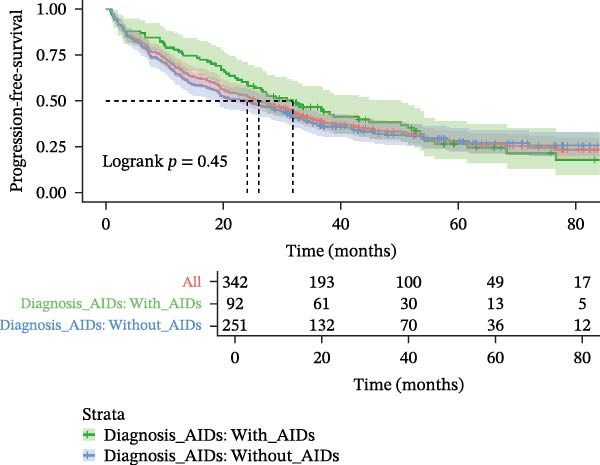
(A)

**Figure:**
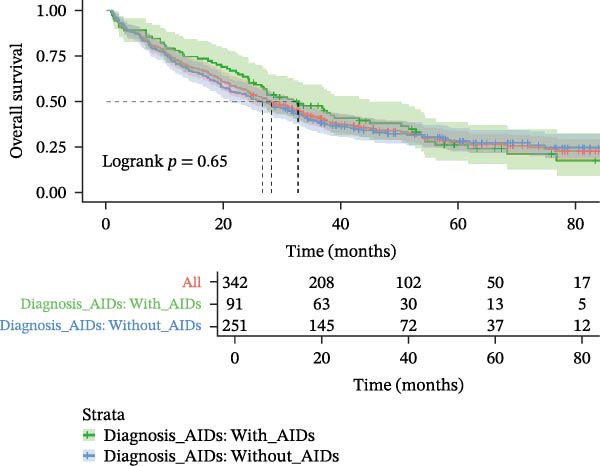
(B)

**Figure:**
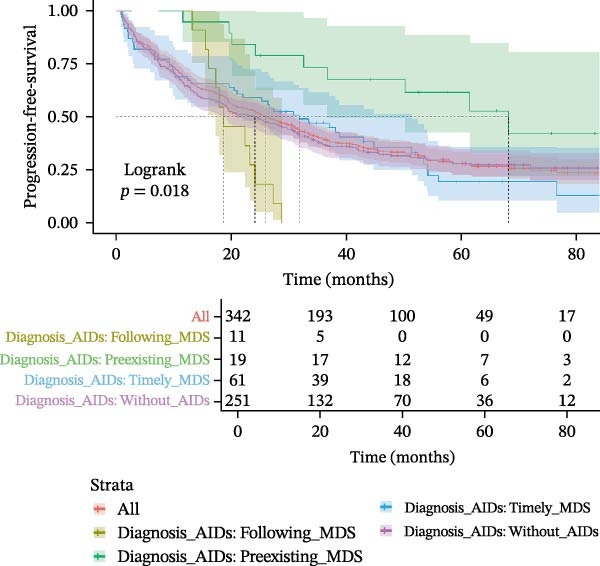
(C)

**Figure:**
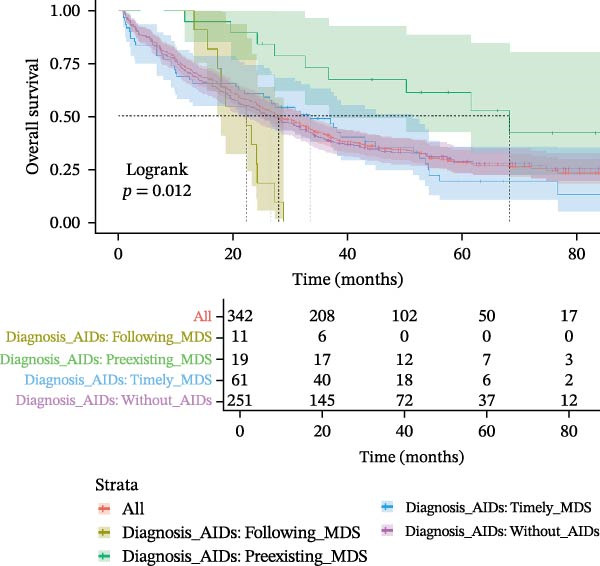
(D)

In univariate analyses, in addition to well‐recognized risk factors for survival (patients’ age, IPSS, IPSS‐R score, karyotype, and bone marrow blasts), pre‐existing and following AIDs also influenced survival. The pre‐existing AIDs was a protective factor (HR = 0.445, IC95% 0.227–0.870, *p* = 0.018), and the following AIDs was a risk of survival (HR = 1.916, IC95% 1.034–3.549, *p* = 0.039). Further multivariate analysis showed the pre‐existing AIDs was an independent protective factor for survival (HR = 0.504, IC95% 0.256–0.993, *p* = 0.048) (Table 3). Therefore, our results suggest that clarifying the occurrence and potential shared pathological mechanisms between AIDs and MDS may contribute to the development of new treatment methods for MDS patients.

**Table 3:** Univariate and multivariate COX proportional hazards models for mortality in MDS patients (*n* = 342).

Variables	Univariate analysis		Multivariate analysis	
	HR (95% CI)	*p* value	HR (95% CI)	*p* value
Age	1.019 (1.009–1.029)	<0.001	1.018 (1.008–1.028)	<0.001
Gender (male vs. female)	1.364 (1.048–1.775)	0.021	1.326 (1.016–1.731)	0.038
Bone marrow blasts, *n* (%)	1.092 (1.067–1.116)	<0.001	1.079 (1.037–1.122)	<0.001
Karyotype				
Intermediate vs. good	1.135 (0.793–1.625)	0.489	—	—
Poor vs. good	1.693 (1.213–2.362)	0.002	1.683 (1.097–2.581)	0.017
IPSS score (≥1.5 vs. <1.5)	2.33 (1.724–2.892)	<0.001	0.697 (0.438–1.109)	0.128
IPSS‐R score (>4.5 vs. ≤4.5)	2.659 (2.032–3.48)	<0.001	1.717 (1.184–2.492)	0.004
With AIDs vs. without AIDs	0.935 (0.702–1.246)	0.648	—	—
Diagnosis AIDs				
Pre‐existing vs. without AIDs	0.445 (0.227–0.870)	0.018	0.504 (0.256–0.993)	0.048
Timely associated vs. without AIDs	1.035 (0.743–1.442)	0.837	—	—
After MDS vs. without AIDs	1.916 (1.034–3.549)	0.039	1.763 (0.936–3.318)	0.079

*Note:* *p* < 0.05 means significance. Pre‐existing MDS (>1 year before MDS), timely associated (1 year before or after MDS diagnosis); following MDS (>1 year after MDS).

Abbreviation: AIDs, autoimmune diseases.

### 3.2. MR Analysis of the Causal Relationship Between MDS and AIDs, As Well As the Mediation Effect of Imbalanced Immune Cells

#### 3.2.1. The Bidirectional Causal Relationship Between MDS and AIDs

Nevertheless, there were many controversies over the existence or origin of the relationship between MDS and AIDs in other observational studies [4–7], and our COX analyses suggested the importance of pre‐existing and following AIDs on MDS survival. However, no studies have demonstrated the causal relationship between MDS and AIDs. MR analysis is an effective tool for investigating a causal relationship between the two diseases from an epidemiogenetic perspective [14]; thus, our study utilized MR to first explore the potential causal relationship between MDS and 10 types of AIDs (common AIDs in MDS and the globe). Among the effects of MDS on AIDs, IVW results showed a potential causal impact of MDS on the risk of SLE (OR = 1.09, 95% CI = 1.02–1.17, *p* = 0.015) and ITP (OR = 1.13, 95% CI = 1.03–1.24, *p* = 0.003) (Figure 3A and Supporting Information 4‐S1A). However, the MR‐Egger method for testing the association between MDS and ITP yielded a direction of effect that was not consistent with IVW, suggesting that this association result is not robust and should be treated with caution (Supporting Information 4‐S1A). When evaluating the causal effect of AIDs on MDS, IVW did not detect a significant causal association (Figure 3B and Supporting Information 4‐S2A). Importantly, our results did not detect significant heterogeneity or horizontal pleiotropy (Supporting Information 4‐S1A). Scatterplots and funnel plots confirmed the stability of the results (Figure 3C,D). Leave‐one‐out analysis showed that the causal effect of MDS on SLE was not affected by any individual SNP (Figure 3E). These results consistently support the existence of a significant causal relationship between genetic prediction results.

Figure 3The bidirectional causal relationships between MDS and AIDs. The forest plots show the causal effect of MDS on AIDs (A) and AIDs on MDS (B). Scatter plots (C), funnel plot (D), and leave‐one‐out plots (E) for the causal association between MDS (exposure) and SLE (outcome). AIDs, autoimmune diseases; SLE, systemic lupus erythematosus.

**Figure:**
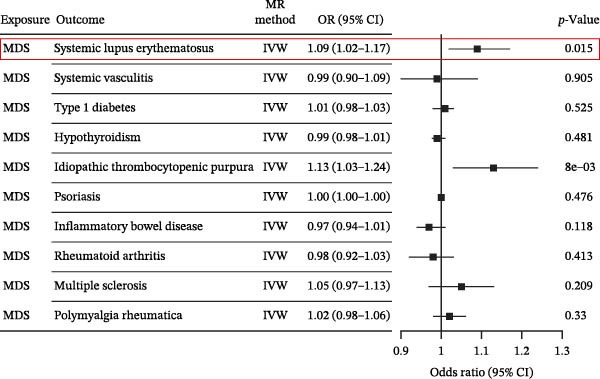
(A)

**Figure:**
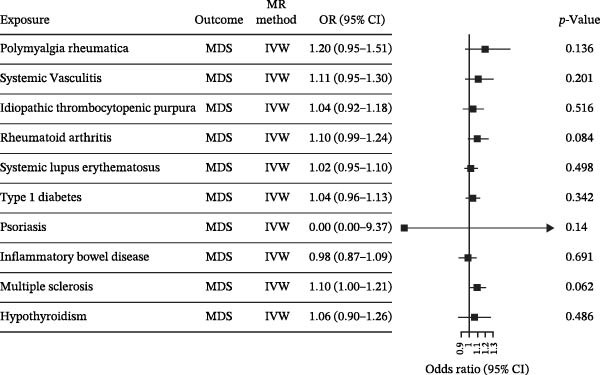
(B)

**Figure:**
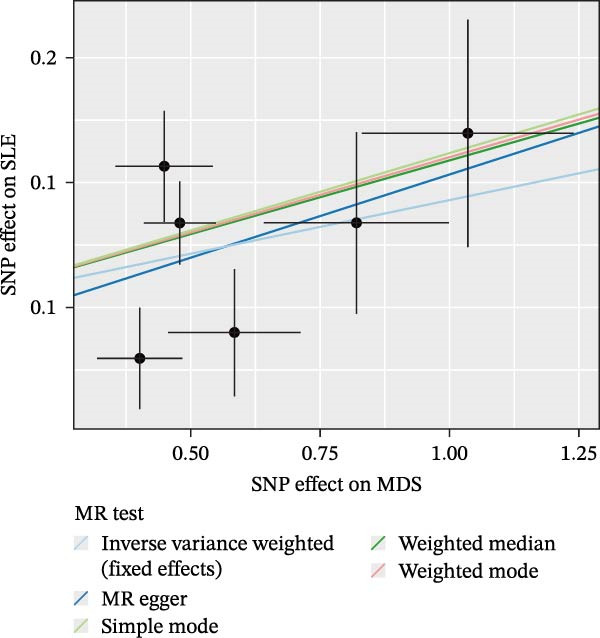
(C)

**Figure:**
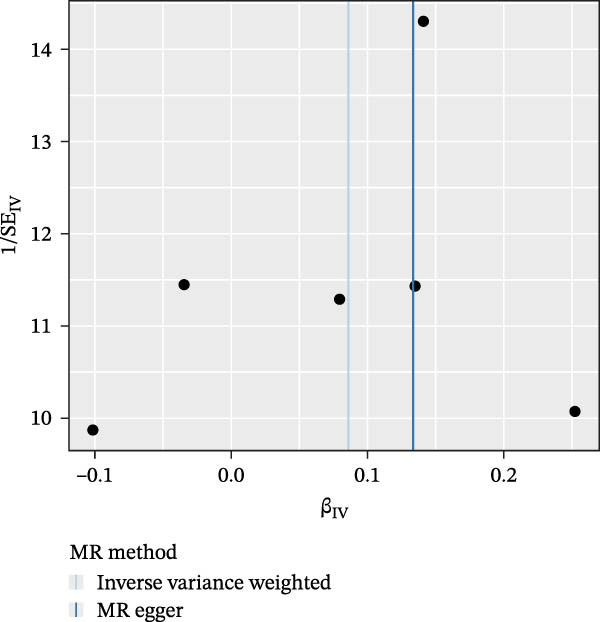
(D)

**Figure:**
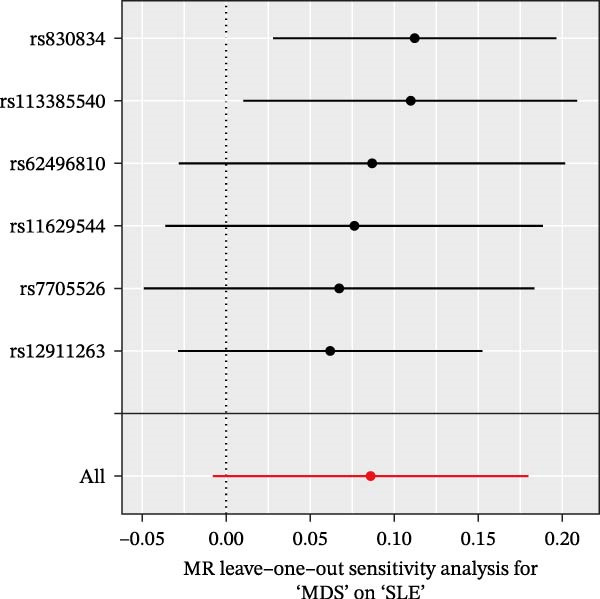
(E)

#### 3.2.2. Mediation Effect of Imbalanced Immune Cells Between MDS and SLE

The mechanisms of MDS and AIDs were often thought to be related to an abnormal immune system [12, 25]. Immune cells, as a crucial important component of immune system homeostasis, have been reported to be involved in the pathophysiologic mechanisms of MDS and AIDs [26–29]. SLE is an independent risk factor for the development of MDS (Figure 4A and Supporting Information 4‐S3A). Subsequently, the causal relationships between MDS and these 15 immune cells were performed by MR analysis. The mediated indirect effects and proportions of naïve CD4+ T‐cells and CD45RA‐CD4+ T‐memory cells in the association between MDS and SLE, and the mediation proportions were 9.0% and 6.9%, respectively (Table 4). These results genetically predicted that naïve CD4+T‐cells and CD45RA‐CD4+T memory cells are potential mediators in MDS‐induced SLE (Figure 4B and Supporting Information 4‐S4A). Since our TSMR results have shown that MDS could increase the risk of SLE, we further explored the key role of 731 types of immune cells in MDS‐induced SLE based on mediated MR analysis. We used TSMR to identify 15 immune cell phenotypes that were significantly causally (FDR < 0.05) associated with SLE.

Figure 4The forest plots for MDS on SLE mediated by immune cells. The forest plots showing the causal effect of MDS on immune cells (A) and immune cells on SLE (B).

**Figure:**
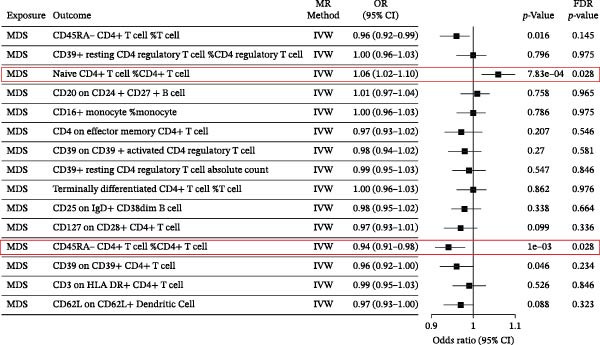
(A)

**Figure:**
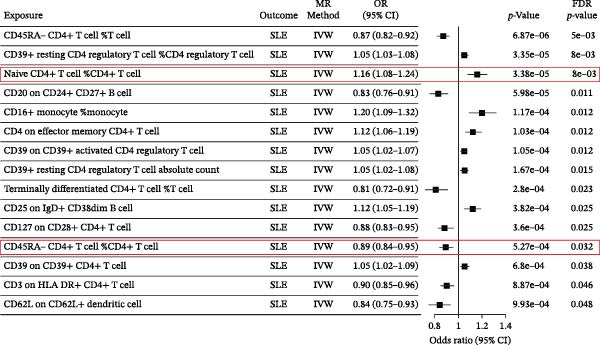
(B)

**Table 4:** Mediation MR analysis for MDS on SLE mediated by immune cells.

Mediator	Direct effect *β*(95% CI)	Mediation effect *β*(95% CI)	Mediation proportion (%)
CD45RA (−)CD4 (+)T‐cell	0.0860 (0.0164, 0.1556)	0.006386381 (0.0011, 0.0117)	6.9120
Naive CD4(+)T‐cell	—	0.008485377 (0.0021, 0.0149)	8.9797

*Note:* “Direct effect” indicates the effect of MDS on SLE; “Mediation effect” indicates the effect of immune cells mediate MDS on SLE; “Mediation proportion” indicates among all mediated effects, the percentage of immune cells‐mediated MDS on SLE.

### 3.3. The Shared Molecular and Cellular Biomarkers for MDS and SLE Were Explored Using Bioinformatic Analysis

#### 3.3.1. Identification of the Crosstalk Genes (CGs) and Its Immune Function in MDS and SLE

Bioinformatics technology can provide a comprehensive analysis of the potential interrelationships in diseases. Since the naïve CD4+ T‐cells and CD45RA‐CD4+ T‐memory cells partially mediated (15.9%) MDS on SLE, the other underlying shared pathology was assessed by bioinformatics analyses. After removing the batch effects of MDS and SLE datasets (Figure 5A,B), differential gene analysis (Figure 5C,D) and WGCNA analysis (Figure 5E–G) were performed. Commonly 76 upregulated differential genes (DEGs), four downregulated DEGs (Figure 5H and Supporting Information 5‐S1A), and three high positively correlated disease‐associated genes using WGCNA were obtained (Figure 5I and Supporting Information 5‐S1A). Considering the crucial roles of WGCNA and differential analysis in disease progression, we have integrated these crosstalk genes (CGs) (Figure 5J) and further performed enrichment analysis. GeneMANIA was used, and the results suggest that these genes are involved in interferon‐related pathways, antiviral‐associated immune responses, and nuclear division‐related regulation (Figure 5K). Further GO and KEGG enrichment analysis consistently suggests associations with signaling pathways involved in antiviral immune responses and other immune regulation, as well as hemoglobin synthesis and myeloid cell chromosomal division mutations (Figure 5L,M). To further screen the genes of the same functional group, we inputted 83 candidate CGs into the String database (Figure 6A) and used Cytoscape to delete the independent genes (Figure 6B,C), followed by the three algorithms of Cytoscape (closeness, degree, and betweenness) to select the core genes (Figure 6D and Supporting Information 5‐S2A). Based on three machine learning algorithms, Lasso regression (Figure 6E,I), support vector machine (Figure 6F,J), and random forest (Figure 6G,K), the most crucial genes were selected in MDS (Figure 6H and Supporting Information 5‐S3A) as well as SLE (Figure 5L and Supporting Information 5‐S3A), and finally identified two diagnostic markers: IFI27 and VSIG4 (Figure 7A).

Figure 5Identification of MDS and SLE shared genes and its functional enrichment. PCA of MDS and SLE original datasets before and after batch‐effect correction (A, B). The heatmap (C) and the volcano plot (D) of differentially expressed genes (DEGs) in the MDS and SLE cohorts. WGCNA analysis of MDS and SLE: scale independence and mean connectivity for soft threshold (β) selection (E), module‐trait relationship heatmap (F), and gene clustering dendrograms (G). The heatmap shows the relationship of module eigengenes with the status of MDS and SLE. A total of 83 key genes (J) were identified by taking the intersection between MDS and SLE via the Venn plot. 76 DEGs were co‐upregulated, 4 DEGs were co‐downregulated (H), and 3 intersecting genes were highly positively correlated with disease in the MDS and SLE via the WGCNA (I). The GeneMANIA analysis (K), GO analysis (L), and KEGG analysis (M) of shared genes.

**Figure:**
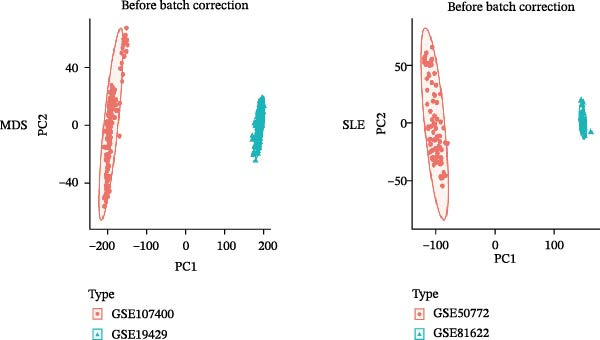
(A)

**Figure:**
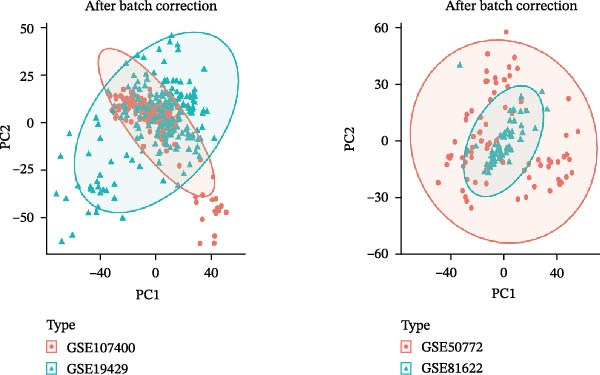
(B)

**Figure:**
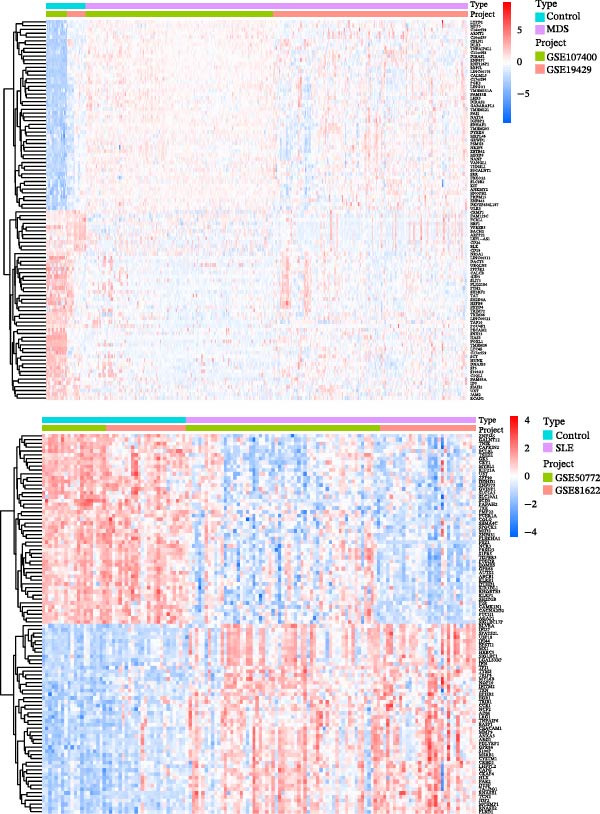
(C)

**Figure:**
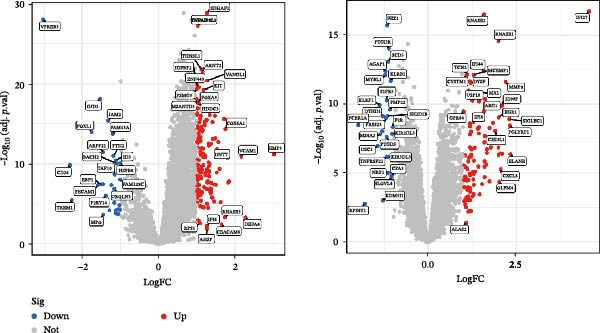
(D)

**Figure:**
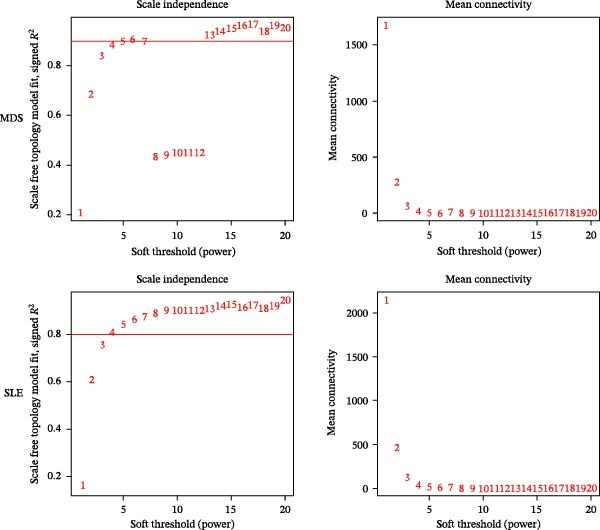
(E)

**Figure:**
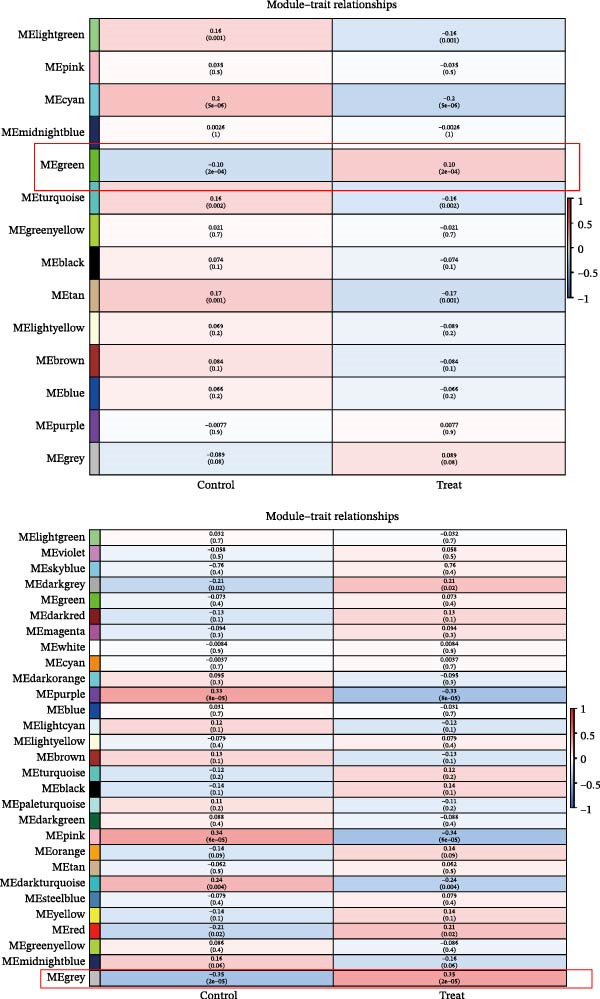
(F)

**Figure:**
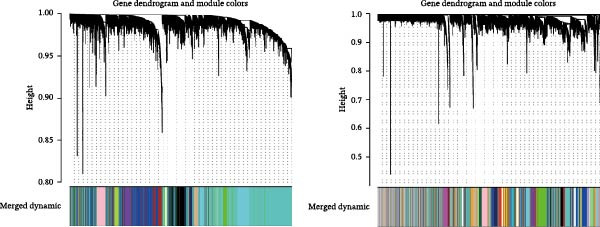
(G)

**Figure:**
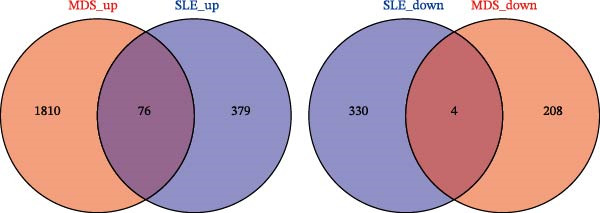
(H)

**Figure:**
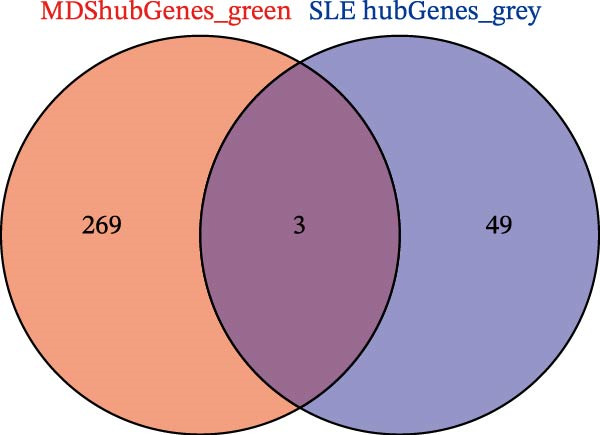
(I)

**Figure:**
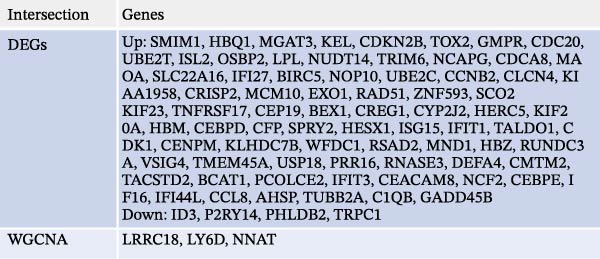
(J)

**Figure:**
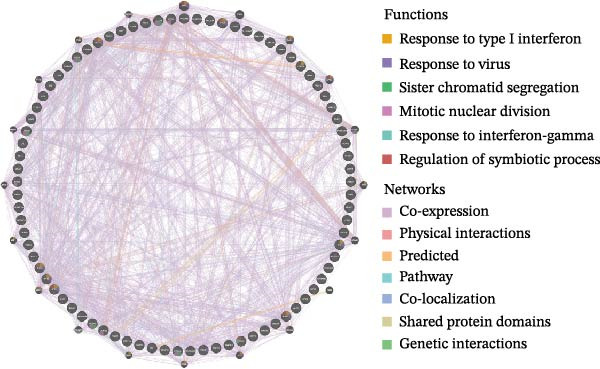
(K)

**Figure:**
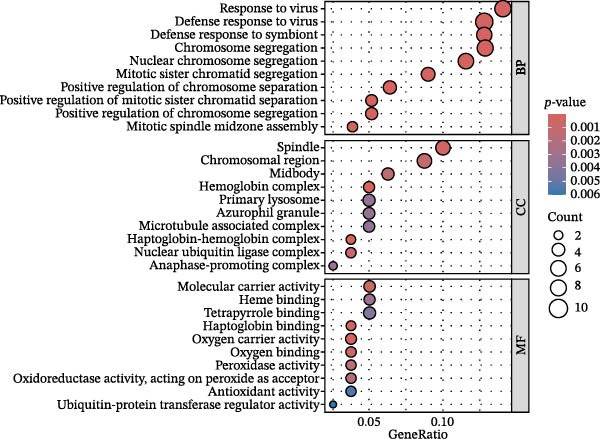
(L)

**Figure:**
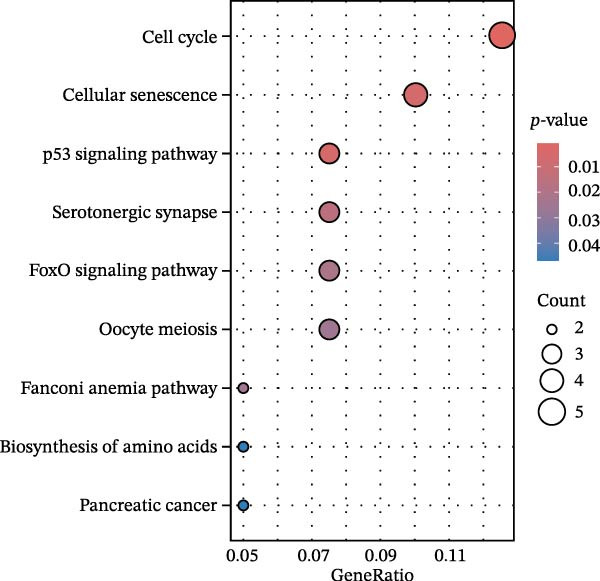
(M)

Figure 6Identification of key shared diagnostic genes. PPI network analysis of genes (A). Cytoscape analysis removes isolated genes (B, C), with the Venn plot showing the 33 intersecting genes of the closeness, betweeness, and degree algorithms (D). LASSO regression analysis of the MDS (E) and SLE (I) cohorts. SVM analysis of the MDS (F) and SLE (J) cohorts. The Random Forest (RF) analysis of the MDS (G) and SLE (K) cohorts. The Venn plot displays intersecting genes among LASSO, SVM, and RF algorithms in the MDS (H) and SLE (L) cohorts.

**Figure:**
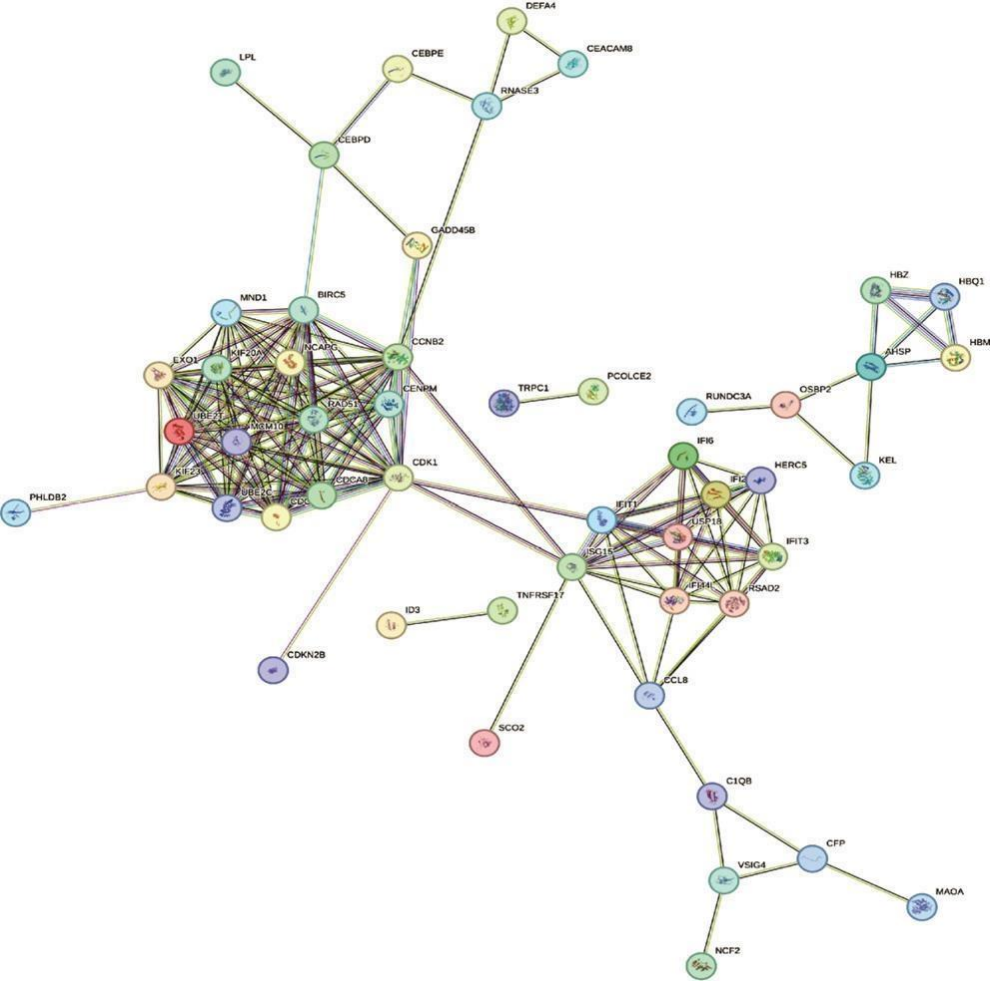
(A)

**Figure:**
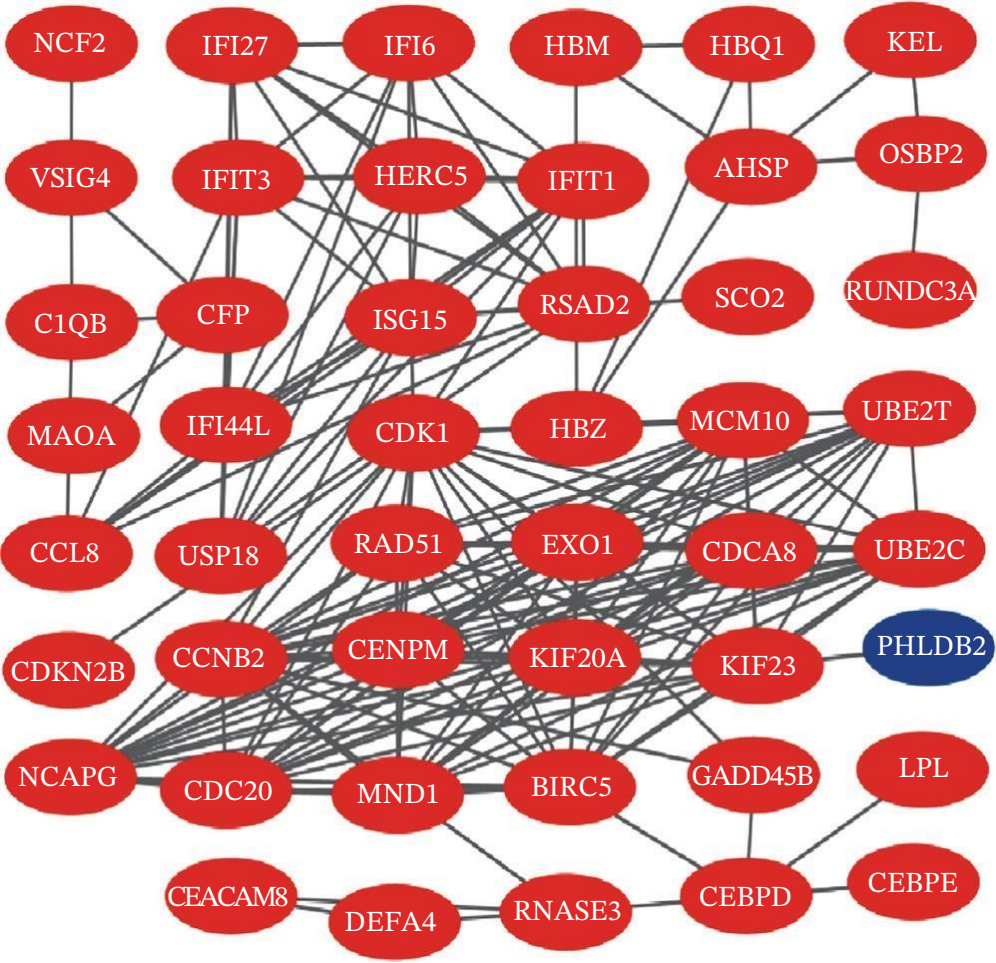
(B)

**Figure:**
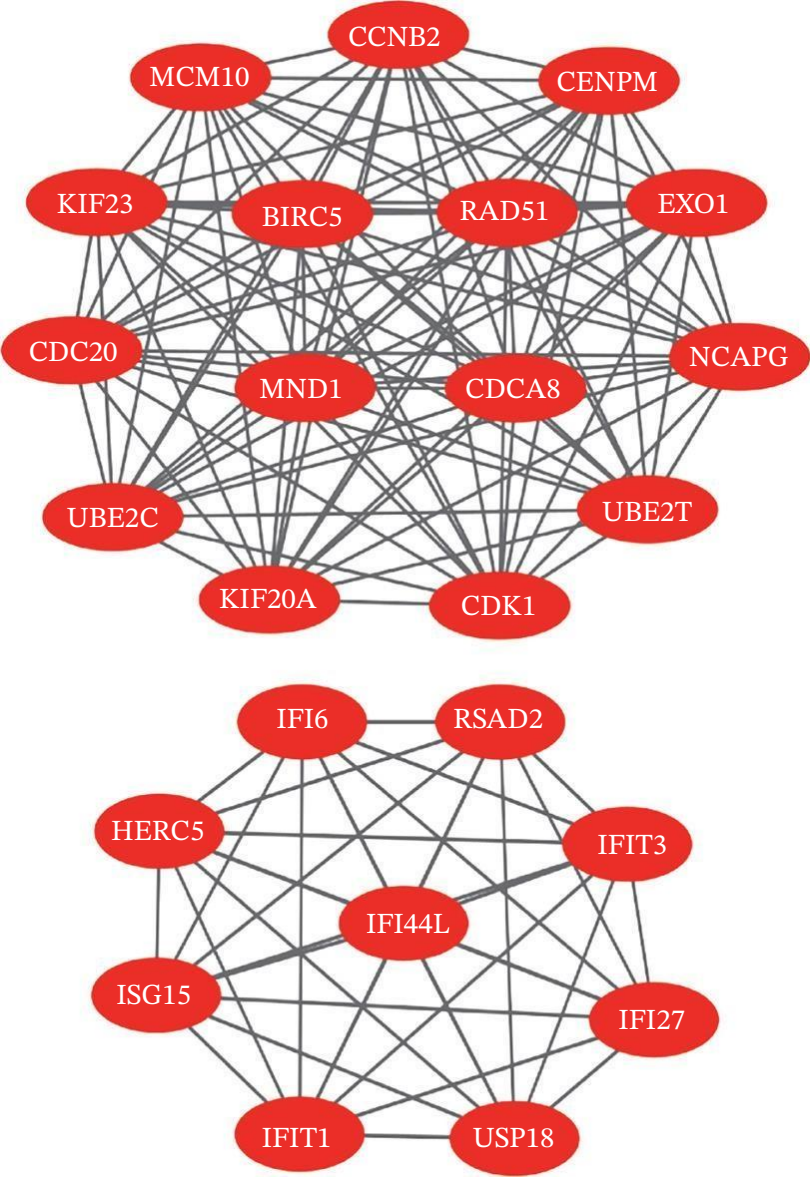
(C)

**Figure:**
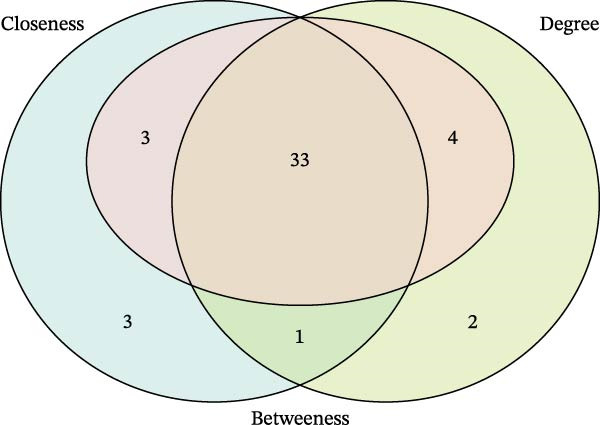
(D)

**Figure:**
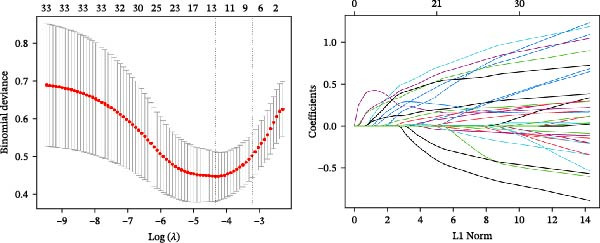
(E)

**Figure:**
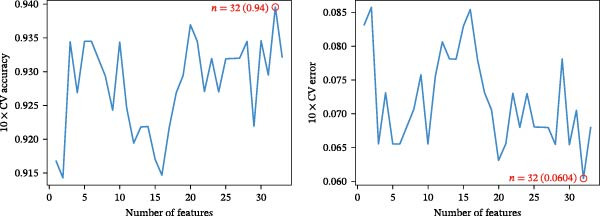
(F)

**Figure:**
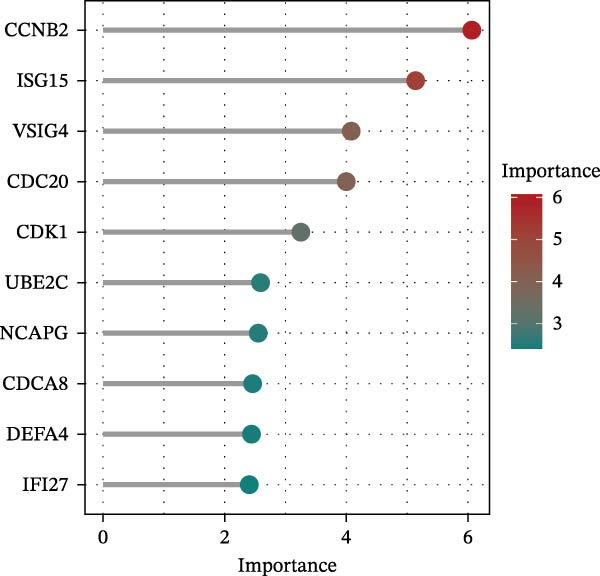
(G)

**Figure:**
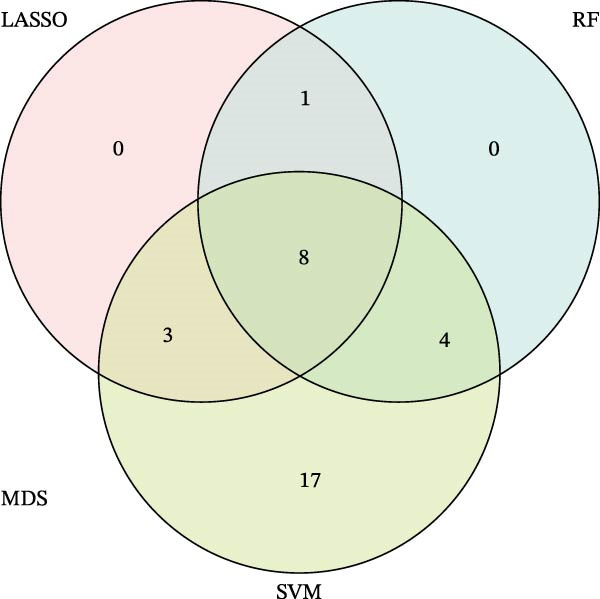
(H)

**Figure:**
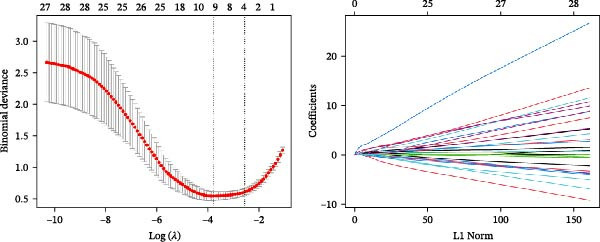
(I)

**Figure:**
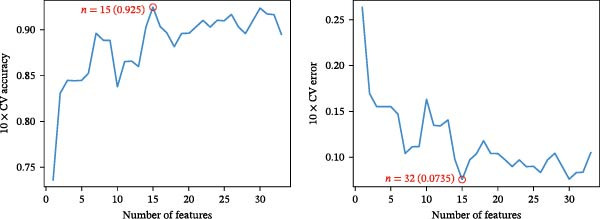
(J)

**Figure:**
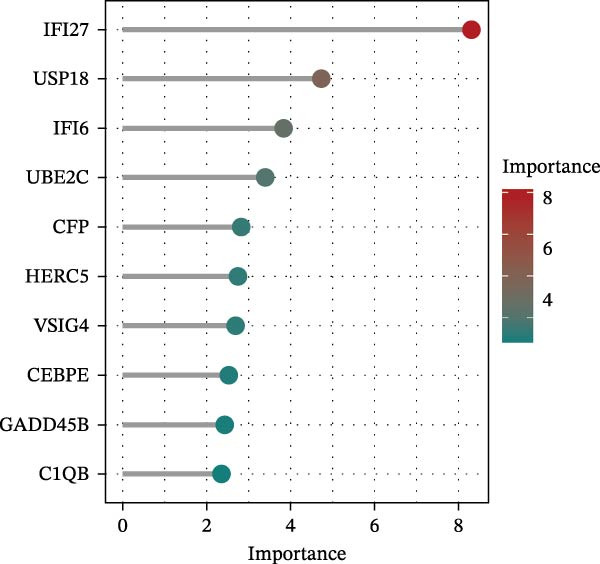
(K)

**Figure:**
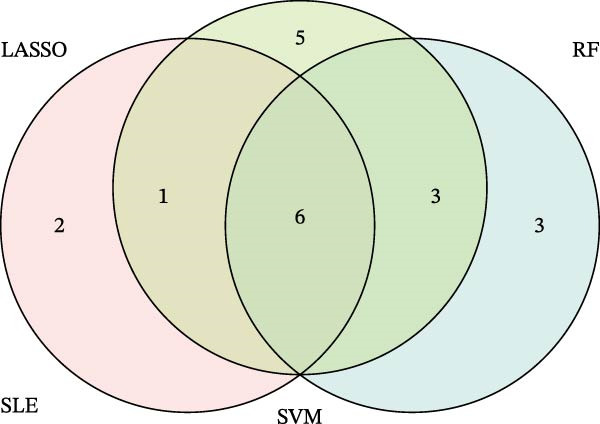
(L)

Figure 7The shared diagnostic genes between MDS and SLE. The Venn plot displaying 2 shared diagnostic genes in MDS and SLE cohorts (A). The expression level of the shared diagnostic biomarkers in MDS and SLE among the training group (B, D) and validation group (C, E). The ROC curves for 2 shared diagnostic biomarkers in the training group and validation group between MDS (F) and SLE (G). GSEA in MDS and SLE (H).

**Figure:**
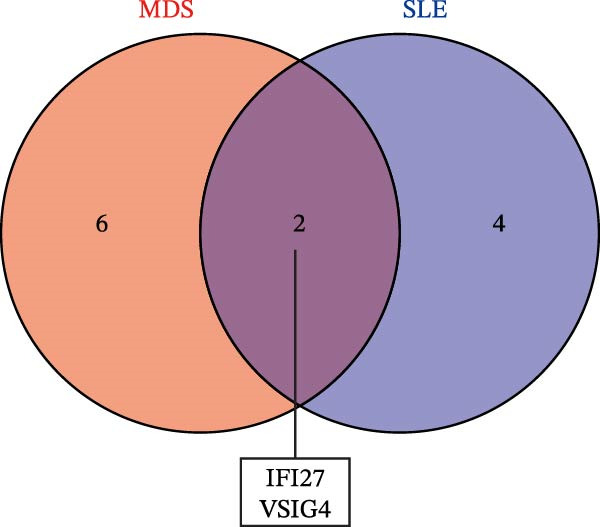
(A)

**Figure:**
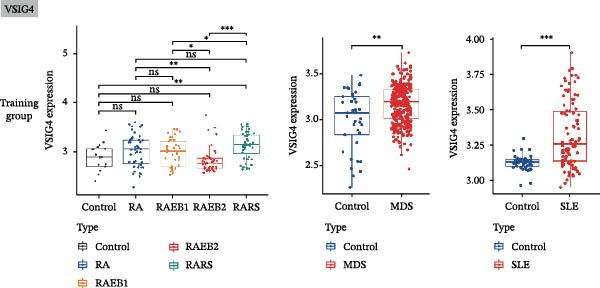
(B)

**Figure:**
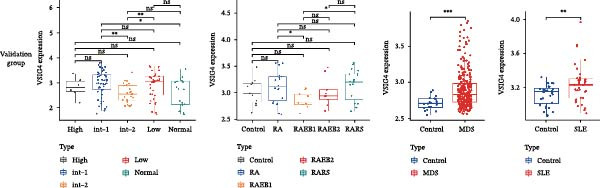
(C)

**Figure:**
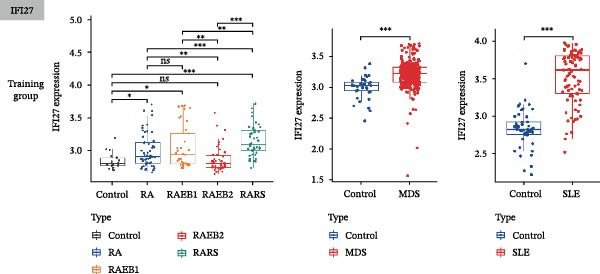
(D)

**Figure:**
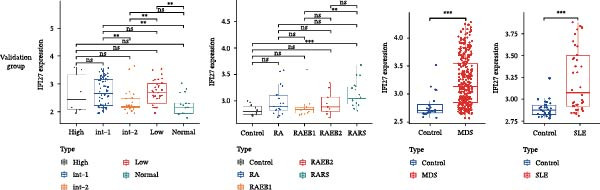
(E)

**Figure:**
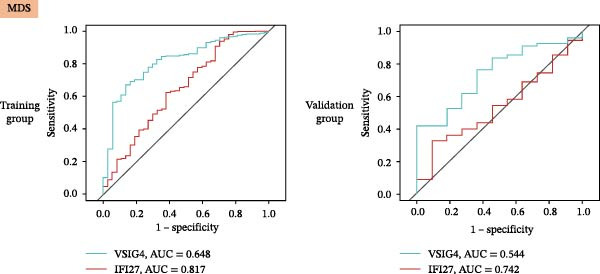
(F)

**Figure:**
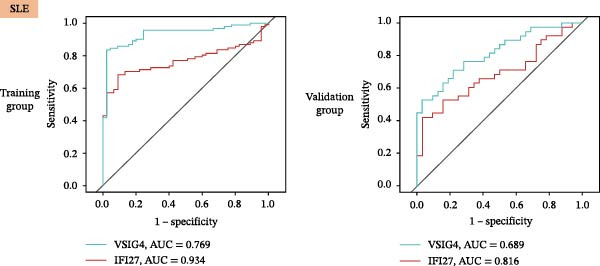
(G)

**Figure:**
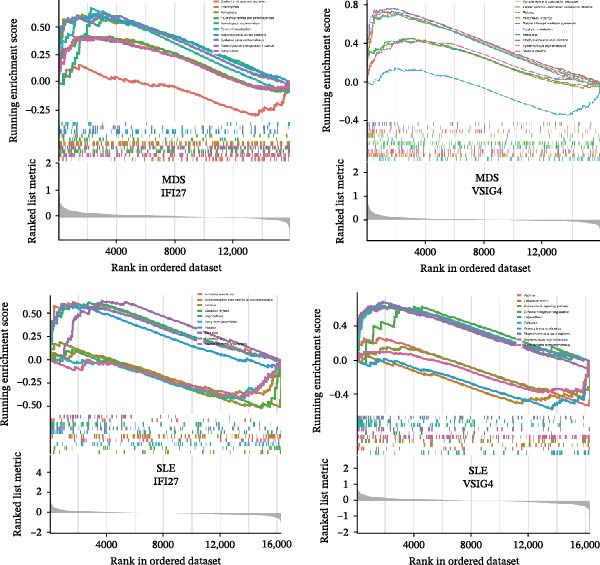
(H)

Furthermore, in the test and validation datasets, the expression of IFI27 and VSIG4 was upregulated in both MDS and SLE compared to the control group (Figure 7B–E). Interestingly, IFI27 and VSIG4 were predominantly differentially expressed in the low‐ and intermediate‐risk type. The diagnostic value of these two genes in different datasets was further assessed using ROC curves, and all the results indicated good disease‐recognizing ability (Figure 7F,G). In addition, GSEA showed that IFI27 and VSIG4 exhibited significant enrichment in the regulation of a variety of cellular and immune response pathways. In MDS, this is mainly associated with ferroptosis and cancer‐related transcriptional dysregulation and is also related to the regulation of SLE, while in SLE, it is primarily focused on TCR and other immune signaling pathways (Figure 7H).

#### 3.3.2. Immune Infiltration Signature in MDS and SLE

Since the imbalance of immune homeostasis was critical for the development of diseases, we utilized CIBERSORT analysis of 22 immune cells to identify different patterns of immune infiltration in MDS and SLE. First, based on the MDS dataset and the SLE dataset, the results showed consistent and significant lymphocyte infiltration compared to the control group, suggesting the presence of immune dysregulation and an inflammatory response in both SLE and MDS (Figure 8A–D). IFI27 and VSIG4 were also significantly correlated with multiple immune cell infiltration levels in SLE and MDS (Figure 8E,F).

Figure 8The immune cell infiltration landscape between MDS and SLE. The stacked histogram of the immune cell proportions in MDS (A) and SLE (C). The violin plot shows the 22 kinds of immune cells between MDS (B) or SLE (D) and the control group. The forest plot of the relationship between shared diagnostic biomarkers and immune cells in MDS (E) and SLE (F).

**Figure:**
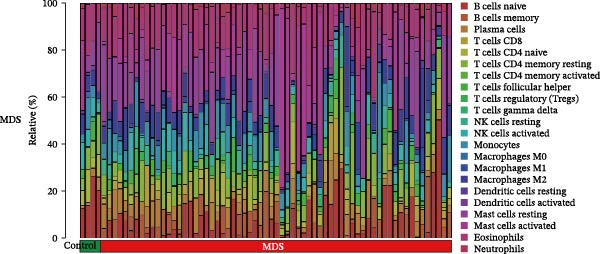
(A)

**Figure:**
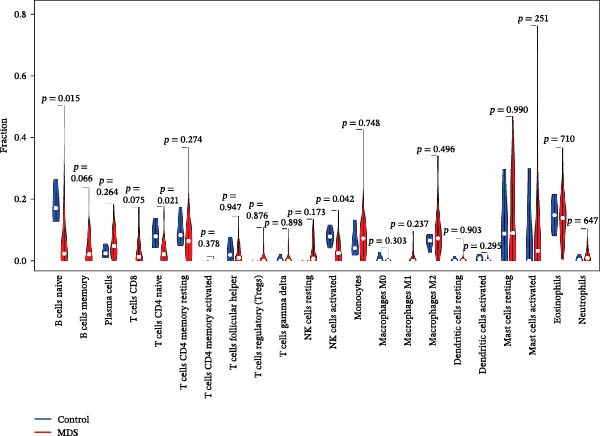
(B)

**Figure:**
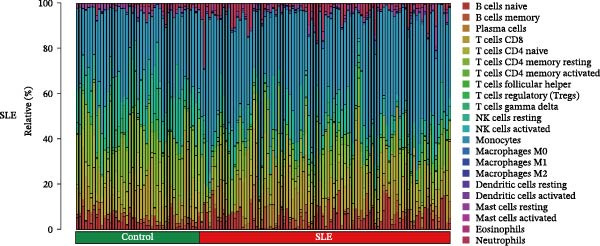
(C)

**Figure:**
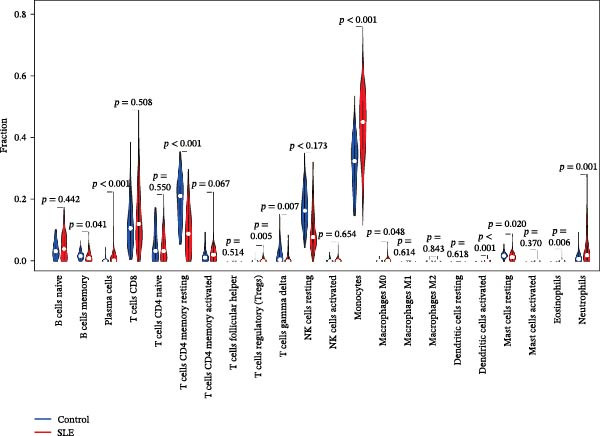
(D)

**Figure:**
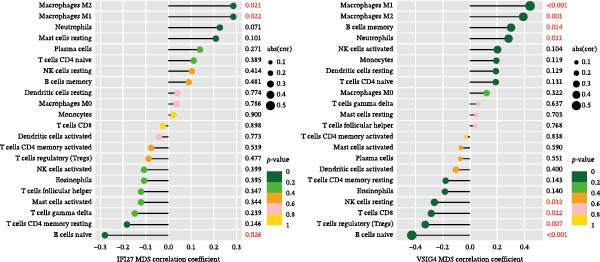
(E)

**Figure:**
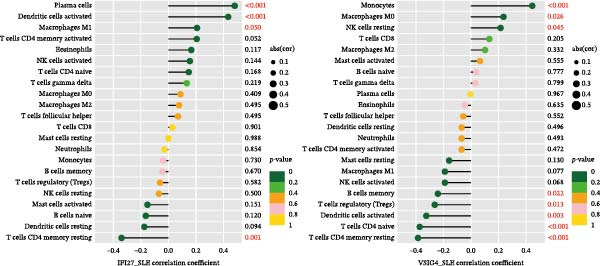
(F)

## 4. Discussion

About 10%–30% MDS patients presented with AIDs, but the development, prognostic impact, and underlying pathophysiological mechanisms between AIDs and MDS remain controversial. Thus, by gaining further insights into the direct relationships and mechanisms of AIDs on MDS, new MDS‐specific targets may be identified. In this study, we provided evidence that there was a significant and direct association between MDS and AIDs. After conducting the multicenter retrospective study based on a Chinese population, we found that AIDs were identified in 26.6% of the MDS patients, and the presence of AIDs appeared to have a lower probability of progression to AML. This is mostly related to the results that MDS patients with AIDs (especially pre‐existing AIDs) are predominantly categorized into the low‐ or intermediate‐1 risk groups, and similar data were found in another study [4]. Additionally, the chronic inflammation associated with AIDs might alter the bone marrow immune microenvironment in a way that is less conducive to the progression of MDS to AML. A higher ratio of Th17/Tregs in low‐risk MDS patients could promote myeloid malignant cell apoptosis [30]. Stem cell mutations (e.g., RUNX1) could accelerate the progression of MDS to AML [31, 32], and upregulation of the level of RUNX1 in MDS also promotes myeloid‐derived suppressor cell (MDSC) expansion [33, 34], which facilitates the immune escape of myeloid malignant cells. Thus, lower levels of MDSCs [35] in the low‐risk MDS group could reduce the accumulation of genetic aberrations, potentially slowing MDS progression.

Furthermore, the following AIDs (>1 year after MDS) appear to be risk factors for MDS survival. Conversely, the pre‐existing AIDs (>1 year before MDS) may serve as an independent protective factor. This is contrary to the conclusions of the French cohort study [13], which may be related to methodological differences in the inclusion of MDS populations and ethnic differences in genetic background, immune response patterns, living environment, and disease susceptibility [36]. These different effects of AIDs on MDS survival are intriguing and warrant further exploration. In addition to a high rate of low‐ and intermediate‐risk MDS patients in the pre‐existing AIDs group, the different immune tolerant status and clonal hematopoiesis could lead to this result. In the relapse or treatment‐resistant MDS group, hematopoietic stem and progenitor cells act as major contributors to induce massive pyroptosis (a more inflammatory and immunogenic form of programed cell death) [37, 38]. This process leads to excessive production of pro‐inflammatory factors and damage‐associated molecular patterns (DAMPs), which contribute to autoimmune pathology [39]. In turn, this hyperinflammatory reaction stimulates the signaling pathways of Toll‐like receptors (TLRs), promoting the expansion of MDSCs, exacerbating bone marrow microenvironmental dysregulation and inflammation [38], leading to a high systemic inflammatory burden, and shortening the survival period in MDS patients [40]. In vivo, Mei et al. [41] demonstrated a significant attenuation in disease progression and an enhancement in survival rates by genetically ablating the IL‐6 gene in a spontaneous mouse model of MDS. In contrast, the immune system of patients with long‐term AIDs may re‐establish tolerance and immune homeostasis [42] or be substantially weakened [43] by both the disease and chronic use of immunosuppressants, in turn reducing the systemic inflammatory burden and extending survival [40]. On the other hand, TET2 mutation is associated with systemic inflammation or AIDs [44–47], predominantly impacting the early stages of MDS initiation [31], and has been reported to be associated with a favorable prognosis in MDS patients [48].

Additionally, we have first utilized bidirectional MR analysis to explore the causal relationships between MDS and various AIDs (common AIDs in MDS and globe). The results showed that MDS increased the risk of developing SLE, although no significant causal relationships were observed in other AIDs. Further, we explored the pathophysiological mechanisms of immune cell imbalance in MDS‐induced SLE and identified potential shared diagnostic biomarkers. Based on MR‐mediated analysis of 731 immune cells, we demonstrated a significant promoting effect of naïve CD4+T‐cells and CD45RA‐CD4+T‐memory cells in MDS‐induced SLE. Previous studies have shown a downregulation of naïve T‐cells [26–28] and an upregulation of CD45RA‐CD4+T memory cells in both MDS and SLE patients [28, 29]. The cellular function also was abnormal. Naïve T‐cells in MDS patients were sustained dysfunctional due to telomerase deficiency [26], which will further lead to T memory cells differentiated from these naïve T‐cells accumulating, potentially inducing or perpetuating SLE [49]. Other immune cells may also be involved in the development of AIDs. NPM1 mutation and STAT3 mutation in MDS lead to the formation of neutrophil extracellular trap (NET) [50, 51] and effector CD8+ T‐cell oligoclonal accumulation [52], respectively, which contribute to autoimmune pathology.

In our study, IFI27 (Interferon Alpha Inducible Protein 27) and VSIG4 (V‐set and immunoglobulin domain containing 4) were identified as shared diagnostic markers for MDS and SLE by bioinformatics analyses; both are upregulated in SLE and MDS (especially in the low‐risk group) patients. IFI27 is an IFN‐stimulated gene involved in the innate immune response [53]; its higher‐level expression could promote the proliferation and invasion of cancer cells [54, 55] and also increase the susceptibility to AIDs [56]. High levels of IFI27 may promote the initiation or progression of SLE by inducing memory B cells, neutrophils, or CD8+ T‐cells PANoptosis [57], and also exacerbate arthritis in patients with RA by regulating macrophage activity via the upregulated PFKFB3‐IFIT27 pathway [58]. VSIG4 is a regulator of macrophage polarization and can promote M2 macrophage polarization and induce the malignant progression of tumor cells when highly expressed [59]. VSIG4 also regulates erythropoiesis in MDS [60], and its rs1044165T allele has been reported as a risk factor for severe functional status in women with RA [61]. In MDS complicated by AIDs, the high expression of IFI27 and VSIG4 may reflect or exacerbate the excessive activation of the immune response, further driving the occurrence and development of both MDS and AIDs, suggesting IFI27 and VSIG4 may hold potential therapeutic value.

The limitations of this study include the retrospective design and limited sample databases. Retrospective analysis cannot definitively determine the origin between MDS and AIDs, although we used MR analysis to address these inherent limitations. Additionally, due to constraints of real‐world data, we were unable to fully obtain information on treatment adherence (e.g., whether patients took medications as prescribed, frequency of missed doses) and details of medication dose adjustments for some patients. These unmeasured factors may introduce bias into the study conclusions: for instance, poor treatment adherence could lead to suboptimal control of AIDs, which in turn may confound the true association between AIDs onset time and MDS prognosis. Due to the insufficient number of genome‐wide significant IVs for MDS and certain AIDs, we relaxed the SNP selection threshold in this study. While this strategy ensured a minimum number of IVs and maintained the feasibility of the analysis, it inevitably increased the likelihood of including weaker or false‐positive associations, which may reduce instrument strength and introduce potential bias in causal inference. To partially mitigate these risks, we calculated the F‐statistics to assess instrument strength and applied multiple sensitivity analyses, which in most cases yielded consistent results without evidence of substantial directional pleiotropy. Nevertheless, we acknowledge that residual bias cannot be completely ruled out. Furthermore, due to the limited sample size and number of valid SNPs in the MDS database, no significant causal associations were found between MDS and other AIDs such as RA, hypothyroidism, or systemic vasculitis. Meanwhile, since neither the published literature nor our study can clearly determine whether IFI27 and VSIG4 are drivers of comorbidity development or merely disease‐associated phenomena, further clarification through basic research or clinical prospective studies will be required in the future. Finally, in the process of exploring the pathophysiological mechanisms between MDS and AIDs using MR and bioinformatics analysis, although we included Asian populations (e.g., GSE154851) in our dataset, the MR data sources were all from European populations; thus, racial differences cannot be ruled out.

## 5. Conclusion

In conclusion, a Chinese population‐based cohort suggested that concomitant AIDs could reduce the probability of progression to AML, especially pre‐existing AIDs, which was considered as an independent protective factor of MDS survival. Furthermore, MR analysis and bioinformatics analysis supported that there was a significant and direct association between MDS and AIDs, and the imbalanced ratio of naïve/memory CD4+ T‐cells and IFI27 and VSIG4 were crucial cells and biomarkers that were shared for MDS and SLE. Those results provided a novel insight into causal relationships, the development of biomarkers, and underlying pathogenesis for MDS with AIDs, providing more accurate treatment guidance.

## Author Contributions

**Qizhao Li and Yujin Guo**: writing – original draft, investigation, validation, formal analysis. **Gao Xiao:** retrospective data collection, validation, formal analysis, edited the manuscript, read final draft. **Yuying Wei**, **Wenjuan Gao**, **Xinyi Zuo**, **Xue Shi**, **Hongyu Zhao**, **and Yuefen Hu**: retrospective data collection. **Johan Rebetz, Elisabeth Semple, Xuejing Song, Li Guo, John W. Semple, and Jun Peng:** edited the manuscript, read final draft. **Gege Feng, Gao Xiao, Qizhao Li, and Shuqian Xu:** provided financial resources, edited the manuscript.

## Funding

This study is supported by the General Program of the National Natural Science Foundation of China (Shuqian Xu, Grant 82470145); National Key Research and Development Program of China (Shuqian Xu, Grant 2024YFC2510500); Cheeloo Young Scholar Program of Shandong University (Shuqian Xu); Shandong Province Natural Science Foundation (Shuqian Xu, Grant ZR2024LSW023; Gao Xiao, Grant ZR2021QH248; and Gege Feng, Grant ZR2023QH070); and by the Shandong Postdoctoral Science Foundation (Qizhao Li, Grant SDZZ‐ZR‐202501042).

## Ethics Statement

The retrospective analysis of this study was based on the hospital’s integrated research platform. Given the retrospective and noninterventional approach, which relied on data obtained from routine clinical care without any additional procedures or risks to the patients, the need for informed consent was waived. The study was approved by the Qilu Hospital Ethics Committee (the leading unit’s ethics committee; KYLL‐202405‐053), which granted a waiver for the requirement of informed consent in accordance with the ethical standards set forth by the Declaration of Helsinki.

## Conflicts of Interest

The authors declare no conflicts of interest.

## Supporting Information

Additional supporting information can be found online in the Supporting Information section.

## Supporting information


**Supporting Information 1** STROBE‐MR‐checklist. The causality and direction of association between MDS and 10 types of AIDs are presented in accordance with the STROBE‐MR checklist.

**Supporting Information 2** Data source. The datasets of GWAS studies for MDS, AIDs traits, and Mediator (the left table), and GEO databases for MDS and SLE (the right table).

**Supporting Information 3** Information of study cohort. The baseline characteristics of the study cohort (AIDs divided into three subtypes).

**Supporting Information 4** MR. The forward MR was conducted to assess the causality of risk factors for AIDs on MDS (S1A). Reverse MR results were also listed and showed the effects of AIDs on MDS (S2A). Additionally, TSMR was performed to identify 15 immune cell phenotypes as the mediator in the causality between SLE (S3A) and MDS (S4A).

**Supporting Information 5** Bioinformatic analysis. The common differential genes (DEGs) and the disease‐associated genes (WGCNA) in MDS and SLE (S1A). Then the final hub genes were identified using three algorithms of cytoscape (S2A) and three machine learning algorithms (S3A).

## Data Availability

All data generated or analyzed during this study are included in this published article and its supporting information files.

## References

[1] Cazzola M. , Myelodysplastic Syndromes, Journal of Medicine. (2020) 383, no. 14, 1358–1374. 10.1056/NEJMra1904794 32997910

[2] Adrianzen-Herrera D. , Sparks A. D. , and Singh R. , et al.Impact of Preexisting Autoimmune Disease on Myelodysplastic Syndromes Outcomes: A Population Analysis, Blood Advances. (2023) 7, no. 22, 6913–6922, 10.1182/bloodadvances.2023011050. 37729616 PMC10685168

[3] Komrokji R. S. , Kulasekararaj A. , and Al Ali N. H. , et al.Autoimmune Diseases and Myelodysplastic Syndromes, American Journal of Hematology. (2016) 91, no. 5, E280–3, 10.1002/ajh.24333, 2-s2.0-84981765568. 26875020

[4] Kim Y.-E. , Ahn S. M. , and Oh J. S. , et al.Incidence of and Risk Factors for Myelodysplastic Syndrome in Patients With Rheumatologic Diseases, Rheumatology. (2024) 63, no. 5, 1305–1312, 10.1093/rheumatology/kead374. 37498607

[5] Anderson L. A. , Pfeiffer R. M. , Landgren O. , Gadalla S. , Berndt S. I. , and Engels E. A. , Risks of Myeloid Malignancies in Patients With Autoimmune Conditions, British Journal of Cancer. (2009) 100, no. 5, 822–828, 10.1038/sj.bjc.6604935, 2-s2.0-61749100610. 19259097 PMC2653768

[6] Kristinsson S. Y. , Björkholm M. , Hultcrantz M. , Derolf Å. R. , Landgren O. , and Goldin L. R. , Chronic Immune Stimulation Might Act as a Trigger for the Development of Acute Myeloid Leukemia or Myelodysplastic Syndromes, Journal of Clinical Oncology. (2011) 29, no. 21, 2897–2903, 10.1200/JCO.2011.34.8540, 2-s2.0-79960709287. 21690473 PMC3138717

[7] Al Ustwani O. , Ford L. A. , and Sait S. J. , et al.Myelodysplastic Syndromes and Autoimmune Diseases—Case Series and Review of Literature, Leukemia Research. (2013) 37, no. 8, 894–899, 10.1016/j.leukres.2013.04.007, 2-s2.0-84879779773. 23692654 PMC3699612

[8] Arinobu Y. , Kashiwado Y. , and Miyawaki K. , et al.Autoimmune Manifestations Associated With Myelodysplastic Syndrome Predict a Poor Prognosis, Medicine. (2021) 100, no. 13, 10.1097/MD.0000000000025406, e25406. 33787649 PMC8021323

[9] Mekinian A. , Grignano E. , and Braun T. , et al.Systemic Inflammatory and Autoimmune Manifestations Associated With Myelodysplastic Syndromes and Chronic Myelomonocytic Leukaemia: A French Multicentre Retrospective Study, Rheumatology. (2016) 55, no. 2, 291–300, 10.1093/rheumatology/kev294, 2-s2.0-84961895848. 26350487

[10] Zhou L. , McMahon C. , and Bhagat T. , et al.Reduced SMAD7 Leads to Overactivation of TGF-Beta Signaling in MDS That Can Be Reversed by a Specific Inhibitor of TGF-Beta Receptor I Kinase, Cancer Research. (2011) 71, no. 3, 955–963, 10.1158/0008-5472.CAN-10-2933, 2-s2.0-79551518231. 21189329 PMC3032816

[11] Muench D. E. , Ferchen K. , and Velu C. S. , et al.SKI Controls MDS-Associated Chronic TGF-β Signaling, Aberrant Splicing, and Stem Cell Fitness, Blood. (2018) 132, no. 21, e24–e34, 10.1182/blood-2018-06-860890, 2-s2.0-85057108976. 30249787 PMC6251005

[12] Peng X. , Zhu X. , and Di T. , et al.The Yin-Yang of Immunity: Immune Dysregulation in Myelodysplastic Syndrome With Different Risk Stratification, Frontiers in Immunology. (2022) 13, 10.3389/fimmu.2022.994053, 994053. 36211357 PMC9537682

[13] Seguier J. , Gelsi-Boyer V. , and Ebbo M. , et al.Autoimmune Diseases in Myelodysplastic Syndrome Favors Patients Survival: A Case Control Study and Literature Review, Autoimmunity Reviews. (2019) 18, no. 1, 36–42, 10.1016/j.autrev.2018.07.009, 2-s2.0-85056229595. 30408583

[14] Smith G. D. and Ebrahim S. , ’Mendelian Randomization’: Can Genetic Epidemiology Contribute to Understanding Environmental Determinants of Disease?, International Journal of Epidemiology. (2003) 32, no. 1, 1–22, 10.1093/ije/dyg070, 2-s2.0-0037322022. 12689998

[15] Arber D. A. , Orazi A. , and Hasserjian R. , et al.The 2016 Revision to the World Health Organization Classification of Myeloid Neoplasms and Acute Leukemia, Blood. (2016) 127, no. 20, 2391–2405, 10.1182/blood-2016-03-643544, 2-s2.0-84974560145. 27069254

[16] Petri M. , Orbai A. M. , and Alarcón G. S. , et al.Derivation and Validation of the Systemic Lupus International Collaborating Clinics Classification Criteria for Systemic Lupus Erythematosus, Arthritis & Rheumatism. (2012) 64, no. 8, 2677–2686, 10.1002/art.34473, 2-s2.0-84864470206. 22553077 PMC3409311

[17] Aletaha D. , Neogi T. , and Silman A. J. , et al.2010 Rheumatoid Arthritis Classification Criteria: An American College of Rheumatology/European League Against Rheumatism Collaborative Initiative, Arthritis & Rheumatism. (2010) 69, no. 9, 1580–1588. 10.1136/ard.2010.138461 20699241

[18] Vitali C. , Bombardieri S. , and Jonsson R. , et al.Classification Criteria for Sjögren’s Syndrome: A Revised Version of the European Criteria Proposed by the American-European Consensus Group, Annals of the Rheumatic Diseases. (2002) 61, no. 6, 554–558, 10.1136/ard.61.6.554, 2-s2.0-0036092482. 12006334 PMC1754137

[19] Rodeghiero F. , Stasi R. , and Gernsheimer T. , et al.Standardization of Terminology, Definitions and Outcome Criteria in Immune Thrombocytopenic Purpura of Adults and Children: Report From an International Working Group, Blood. (2009) 113, no. 11, 2386–2393, 10.1182/blood-2008-07-162503, 2-s2.0-64049085194. 19005182

[20] Barrett T. , Wilhite S. E. , and Ledoux P. , et al.NCBI GEO: Archive for Functional Genomics Data Sets—Update, Nucleic Acids Research. (2013) D991–D995. 23193258 10.1093/nar/gks1193 PMC3531084

[21] Feng Z. , Liao M. , Guo X. , Li L. , and Zhang L. , Effects of Immune Cells in Mediating the Relationship Between Gut Microbiota and Myelodysplastic Syndrome: A Bidirectional Two-Sample, Two-Step Mendelian Randomization Study, Discover Oncology. (2024) 15, no. 1, 10.1007/s12672-024-01061-6, 199. 38819469 PMC11143100

[22] Shan M. , Xu L. , and Yang W. , et al.Identification of Common Hub Genes and Construction of Immune Regulatory Networks in Aplastic Anemia, Myelodysplastic Syndromes, and Acute Myeloid Leukemia, Frontiers in Immunology. (2025) 16, 10.3389/fimmu.2025.1547289, 1547289. 40406144 PMC12095185

[23] Chen Z. , Guo Y. , and Sun H. , et al.Exploration of the Causal Associations Between Circulating Inflammatory Proteins, Immune Cells, and Neuromyelitis Optica Spectrum Disorder: A Bidirectional Mendelian Randomization Study and Mediation Analysis, Frontiers in Aging Neuroscience. (2024) 16, 10.3389/fnagi.2024.1394738, 1394738. 38737586 PMC11088236

[24] Chen Z. , Sun H. , and Zhang W. , et al.Exploring Correlations Between Immune Cell Phenotypes and the Risk of Epilepsy: A Bidirectional Mendelian Randomization Study, Epilepsy & Behavior. (2024) 157, 109896. 38905914 10.1016/j.yebeh.2024.109896

[25] Fozza C. , La Nasa G. , and Caocci G. , The Yin and Yang of Myelodysplastic Syndromes and Autoimmunity: The Paradox of Autoimmune Disorders Responding to Therapies Specific for MDS, Critical Reviews in Oncology/Hematology. (2019) 142, 51–57, 10.1016/j.critrevonc.2019.07.018, 2-s2.0-85069947313. 31376677

[26] Yang L. , Mailloux A. , and Rollison D. E. , et al.Naive T-Cells in Myelodysplastic Syndrome Display Intrinsic Human Telomerase Reverse Transcriptase (hTERT) Deficiency, Leukemia. (2013) 27, no. 4, 897–906, 10.1038/leu.2012.300, 2-s2.0-84876151219. 23072779 PMC4346223

[27] Perez R. K. , Gordon M. G. , and Subramaniam M. , et al.Single-Cell RNA-Seq Reveals Cell Type-Specific Molecular and Genetic Associations to Lupus, Science. (2022) 376, no. 6589, 10.1126/science.abf1970, eabf1970. 35389781 PMC9297655

[28] Zou J. X. , Rollison D. E. , and Boulware D. , et al.Altered Naive and Memory CD4+ T-Cell Homeostasis and Immunosenescence Characterize Younger Patients With Myelodysplastic Syndrome, Leukemia. (2009) 23, no. 7, 1288–1296, 10.1038/leu.2009.14, 2-s2.0-67650924504. 19282834 PMC3252820

[29] Han B. K. , White A. M. , Dao K. H. , Karp D. R. , Wakeland E. K. , and Davis L. S. , Increased Prevalence of Activated CD70+CD4+ T Cells in the Periphery of Patients With Systemic Lupus Erythematosus, Lupus. (2005) 14, no. 8, 598–606, 10.1191/0961203305lu2171oa, 2-s2.0-24744445241. 16175931

[30] Kordasti S. Y. , Afzali B. , and Lim Z. , et al.IL-17-Producing CD4 ^+^ T Cells, Pro-Inflammatory Cytokines and Apoptosis are Increased in Low Risk Myelodysplastic Syndrome, British Journal of Haematology. (2009) 145, no. 1, 64–72, 10.1111/j.1365-2141.2009.07593.x, 2-s2.0-61849168098. 19210506

[31] Chen J. , Kao Y.-R. , and Sun D. , et al.Myelodysplastic Syndrome Progression to Acute Myeloid Leukemia at the Stem Cell Level, Nature Medicine. (2019) 25, no. 1, 103–110, 10.1038/s41591-018-0267-4, 2-s2.0-85058062330. PMC6436966 30510255

[32] Jain A. G. , Ball S. , and Aguirre L. , et al.Patterns of Lower Risk Myelodysplastic Syndrome Progression: Factors Predicting Progression to High-Risk Myelodysplastic Syndrome and Acute Myeloid Leukemia, Haematologica. (2024) 109, no. 7, 2157–2164, 10.3324/haematol.2023.283661. 38299605 PMC11215361

[33] Li S. , Li F. , and Xu L. , et al.TLR2 Agonist Promotes Myeloid-Derived Suppressor Cell Polarization via Runx1 in Hepatocellular Carcinoma, International Immunopharmacology. (2022) 111, 10.1016/j.intimp.2022.109168, 109168. 35998504

[34] Thakuri B. K. C. , Zhang J. , and Zhao J. , et al.HCV-Associated Exosomes Upregulate RUNXOR and RUNX1 Expressions to Promote MDSC Expansion and Suppressive Functions Through STAT3-miR124 Axis, Cells. (2020) 9, no. 12, 10.3390/cells9122715, 2715. 33353065 PMC7766103

[35] Syed K. , Naguib S. , Liu Z.-J. , Cimmino L. , and Yang F.-C. , Novel Combinations to Improve Hematopoiesis in Myelodysplastic Syndrome, Stem Cell Research & Therapy. (2020) 11, no. 1, 10.1186/s13287-020-01647-1, 132. 32197634 PMC7083030

[36] Saiganesh A. , Hales B. J. , Li Y. , Holt P. G. , Souëf P. N. L. , and Zhang G. , A Marked Shift in Innate and Adaptive Immune Response in Chinese Immigrants Living in a Western Environment, Allergy. (2018) 73, no. 10, 2092–2094, 10.1111/all.13531, 2-s2.0-85050638973. 29935021

[37] Shastri A. , Will B. , Steidl U. , and Verma A. , Stem and Progenitor Cell Alterations in Myelodysplastic Syndromes, Blood. (2017) 129, no. 12, 1586–1594, 10.1182/blood-2016-10-696062, 2-s2.0-85016256866. 28159737 PMC5364336

[38] Barreyro L. , Chlon T. M. , and Starczynowski D. T. , Chronic Immune Response Dysregulation in MDS Pathogenesis, Blood. (2018) 132, no. 15, 1553–1560, 10.1182/blood-2018-03-784116, 2-s2.0-85054733047. 30104218 PMC6182269

[39] Basiorka A. A. , McGraw K. L. , and Eksioglu E. A. , et al.The NLRP3 Inflammasome Functions as a Driver of the Myelodysplastic Syndrome Phenotype, Blood. (2016) 128, no. 25, 2960–2975, 10.1182/blood-2016-07-730556, 2-s2.0-85010214978. 27737891 PMC5179338

[40] Nielsen A. B. , Hansen J. W. , and Ørskov A. D. , et al.Inflammatory Cytokine Profiles Do Not Differ Between Patients With Idiopathic Cytopenias of Undetermined Significance and Myelodysplastic Syndromes, HemaSphere. (2022) 6, no. 5, 10.1097/HS9.0000000000000713, e0713. 35495296 PMC9038488

[41] Mei Y. , Ren K. , and Liu Y. , et al.Bone Marrow-Confined IL-6 Signaling Mediates the Progression of Myelodysplastic Syndromes to Acute Myeloid Leukemia, Journal of Clinical Investigation. (2022) 132, no. 17, 10.1172/JCI152673, e152673. 35900794 PMC9435651

[42] Shuai Z. , Zheng S. , Wang K. , Wang J. , Leung P. S. C. , and Xu B. , Reestablish Immune Tolerance in Rheumatoid Arthritis, Frontiers in Immunology. (2022) 13, 10.3389/fimmu.2022.1012868, 1012868. 36248797 PMC9561630

[43] Swart J. F. , Delemarre E. M. , and van Wijk F. , et al.Haematopoietic Stem Cell Transplantation for Autoimmune Diseases, Nature Reviews Rheumatology. (2017) 13, no. 4, 244–256, 10.1038/nrrheum.2017.7, 2-s2.0-85013465395. 28228650

[44] Oh Y. J. , Shin D. Y. , and Hwang S. M. , et al.Mutation of Ten-Eleven Translocation-2 Is Associated With Increased Risk of Autoimmune Disease in Patients With Myelodysplastic Syndrome, The Korean Journal of Internal Medicine. (2020) 35, no. 2, 457–464, 10.3904/kjim.2018.247. 31640337 PMC7061008

[45] Pandey S. P. , Bender M. J. , and McPherson A. C. , et al.Tet2 Deficiency Drives Liver Microbiome Dysbiosis Triggering Tc1 Cell Autoimmune Hepatitis, Cell Host & Microbe. (2022) 30, no. 7, 1003–1019.e10, 10.1016/j.chom.2022.05.006. 35658976 PMC9841318

[46] López-Nevado M. , Ortiz-Martín J. , and Serrano C. , et al.Novel Germline TET2 Mutations in Two Unrelated Patients With Autoimmune Lymphoproliferative Syndrome-Like Phenotype and Hematologic Malignancy, Journal of Clinical Immunology. (2023) 43, no. 1, 165–180, 10.1007/s10875-022-01361-y. 36066697

[47] Fattizzo B. , Levati G. V. , and Giannotta J. A. , et al.Low-Risk Myelodysplastic Syndrome Revisited: Morphological, Autoimmune, and Molecular Features as Predictors of Outcome in a Single Center Experience, Frontiers in Oncology. (2022) 12, 10.3389/fonc.2022.795955, 795955. 35392224 PMC8980524

[48] Kosmider O. , Gelsi-Boyer V. , and Cheok M. , et al.TET2 Mutation Is an Independent Favorable Prognostic Factor in Myelodysplastic Syndromes (MDSs), Blood. (2009) 114, no. 15, 3285–3291, 10.1182/blood-2009-04-215814, 2-s2.0-70350438115. 19666869

[49] Fritsch R. D. , Shen X. , and Illei G. G. , et al.Abnormal Differentiation of Memory T Cells in Systemic Lupus Erythematosus, Arthritis & Rheumatism. (2006) 54, no. 7, 2184–2197, 10.1002/art.21943, 2-s2.0-33745906975. 16802356

[50] Tripodo C. , Burocchi A. , and Piccaluga P. P. , et al.Persistent Immune Stimulation Exacerbates Genetically Driven Myeloproliferative Disorders via Stromal Remodeling, Cancer Research. (2017) 77, no. 13, 3685–3699, 10.1158/0008-5472.CAN-17-1098, 2-s2.0-85023752947. 28536276

[51] Lee K. H. , Kronbichler A. , and Park D. D. , et al.Neutrophil Extracellular Traps (NETs) in Autoimmune Diseases: A Comprehensive Review, Autoimmunity Reviews. (2017) 16, no. 11, 1160–1173, 10.1016/j.autrev.2017.09.012, 2-s2.0-85030761499. 28899799

[52] Masle-Farquhar E. , Jackson K. J. L. , and Peters T. J. , et al.STAT3 Gain-of-Function Mutations Connect Leukemia With Autoimmune Disease by Pathological NKG2D(hi) CD8+ T Cell Dysregulation and Accumulation, Immunity. (2022) 55, no. 12, 2386–2404.e8, 10.1016/j.immuni.2022.11.001. 36446385

[53] Zhao X. , Zhang L. , and Wang J. , et al.Identification of Key Biomarkers and Immune Infiltration in Systemic Lupus Erythematosus by Integrated Bioinformatics Analysis, Journal of Translational Medicine. (2021) 19, no. 1, 10.1186/s12967-020-02698-x, 35. 33468161 PMC7814551

[54] Wang H. , Qiu X. , Lin S. , Chen X. , Wang T. , and Liao T. , Knockdown of IFI27 Inhibits Cell Proliferation and Invasion in Oral Squamous Cell Carcinoma, World Journal of Surgical Oncology. (2018) 16, no. 1, 10.1186/s12957-018-1371-0, 2-s2.0-85045213769, 64. 29580248 PMC5870725

[55] Sagou K. , Sato Y. , and Okuno Y. , et al.Epstein-Barr Virus Lytic Gene BNRF1 Promotes B-Cell Lymphomagenesis via IFI27 Upregulation, PLOS Pathogens. (2024) 20, no. 2, 10.1371/journal.ppat.1011954, e1011954. 38300891 PMC10833513

[56] O’Hanlon T. P. , Rider L. G. , and Gan L. , et al.Gene Expression Profiles From Discordant Monozygotic Twins Suggest That Molecular Pathways Are Shared Among Multiple Systemic Autoimmune Diseases, Arthritis Research & Therapy. (2011) 13, no. 2, 10.1186/ar3330, 2-s2.0-79955109537, R69. 21521520 PMC3132064

[57] Sun W. , Li P. , and Wang M. , et al.Molecular Characterization of PANoptosis-Related Genes With Features of Immune Dysregulation in Systemic Lupus Erythematosus, Clinical Immunology. (2023) 253, 10.1016/j.clim.2023.109660, 109660. 37295541

[58] Zhu X. , Zhou X. , and Li S. , et al.PFKFB3 Decreases α-Ketoglutarate Production While Partial PFKFB3 Knockdown in Macrophages Ameliorates Arthritis in Tumor Necrosis Factor-Transgenic Mice, International Immunopharmacology. (2025) 148, 10.1016/j.intimp.2025.114102, 114102. 39870011

[59] Liu J. , Zhang W. , and Chen L. , et al.VSIG4 Promotes Tumour-Associated Macrophage M2 Polarization and Immune Escape in Colorectal Cancer via Fatty Acid Oxidation Pathway, Clinical and Translational Medicine. (2025) 15, no. 5, 10.1002/ctm2.70340, e70340. 40405491 PMC12098961

[60] Pellagatti A. , Jädersten M. , and Forsblom A. M. , et al.Lenalidomide Inhibits the Malignant Clone and Up-Regulates the SPARC Gene Mapping to the Commonly Deleted Region in 5q- Syndrome Patients, Proceedings of the National Academy of Sciences. (2007) 104, no. 27, 11406–11411, 10.1073/pnas.0610477104, 2-s2.0-34547474047. PMC1892786 17576924

[61] Andrade F. A. , Eibofner I. G. , and Pieczarka C. , et al.Impact of *VSIG4* Gene Polymorphisms on Susceptibility and Functional Status of Rheumatoid Arthritis, International Journal of Immunogenetics. (2021) 48, no. 3, 260–265, 10.1111/iji.12533. 33645007

